# General Anesthesia During Neurodevelopment Reduces Autistic Behavior in Adult BTBR Mice, a Murine Model of Autism

**DOI:** 10.3389/fncel.2021.772047

**Published:** 2021-11-29

**Authors:** Jianchen Cui, Jiho Park, Xianshu Ju, Yulim Lee, Boohwi Hong, Jeonghoon Ahn, Yoon Hee Kim, Youngkwon Ko, Seok-Hwa Yoon, Chaeseong Lim, Sun Yeul Lee, Sung-Oh Huh, Jun Young Heo, Woosuk Chung

**Affiliations:** ^1^Department of Medical Science, Chungnam National University School of Medicine, Daejeon, South Korea; ^2^Department of Biochemistry, Chungnam National University School of Medicine, Daejeon, South Korea; ^3^Infection Control Convergence Research Center, Chungnam National University School of Medicine, Daejeon, South Korea; ^4^Department of Anesthesiology, The First People’s Hospital of Yunnan Province, The Affiliated Hospital of Kunming University of Science and Technology, Kunming, China; ^5^Brain Korea 21 FOUR Project for Medical Science, Chungnam National University, Daejeon, South Korea; ^6^Department of Anesthesiology and Pain Medicine, Chungnam National University Hospital, Daejeon, South Korea; ^7^Department of Anesthesiology and Pain Medicine, Chungnam National University School of Medicine, Daejeon, South Korea; ^8^Department of Pharmacology, College of Medicine, Institute of Natural Medicine, Hallym University, Chuncheon, South Korea

**Keywords:** autism, anesthesia, BDNF (brain derived neurotrophic factor), BTBR, E/I imbalance, mitochondrial respiration

## Abstract

Preclinical studies suggest that repeated exposure to anesthetics during a critical period of neurodevelopment induces long-term changes in synaptic transmission, plasticity, and behavior. Such changes are of great concern, as similar changes have also been identified in animal models of neurodevelopmental disorders (NDDs) such as autism. Because of overlapping synaptic changes, it is also possible that anesthetic exposures have a more significant effect in individuals diagnosed with NDDs. Thus, we evaluated the effects of early, multiple anesthetic exposures in BTBR mice, an inbred strain that displays autistic behavior. We discovered that three cycles of sevoflurane anesthesia (2.5%, 1 h) with 2-h intervals between each exposure in late postnatal BTBR mice did not aggravate, but instead improved pathophysiological mechanisms involved with autistic behavior. Sevoflurane exposures restored E/I balance (by increasing inhibitory synaptic transmission), and increased mitochondrial respiration and BDNF signaling in BTBR mice. Most importantly, such changes were associated with reduced autistic behavior in BTBR mice, as sociability was increased in the three-chamber test and repetitive behavior was reduced in the self-grooming test. Our results suggest that anesthetic exposures during neurodevelopment may affect individuals diagnosed with NDDs differently.

## Introduction

Although recent clinical studies agree that anesthetic exposure in young children does not affect general cognition, several studies have suggested significant changes in specific developmental domains such as motor and social performance (Warner et al., 2018; Walkden et al., 2020; Ing et al., 2021). Also, the neurotoxic effects of prolonged or multiple anesthetic exposures also remain undetermined. Preclinical animal studies, including those in rodents, sheep and non-human primates, strongly suggest that multiple anesthetic exposures, unlike a single exposure, may affect neurodevelopment (Olutoye et al., 2016; Chung et al., 2017b; Raper et al., 2018; Ju et al., 2020a; Neudecker et al., 2021). Recent clinical studies also support a distinction between single and multiple anesthetic exposures, with the latter suggested to have long-term negative consequences (Hu et al., 2017; Warner et al., 2018; Zaccariello et al., 2019).

Our group recently reported similar results of exposing postnatal day 16, 17 (PND16/17) mice to sevoflurane. Unlike a single, short sevoflurane exposure (Chung et al., 2017a), multiple sevoflurane exposures induced long-lasting changes in synaptic transmission, plasticity, and behavior (Ju et al., 2020a). Such long-lasting synaptic changes after multiple anesthetic exposures are of great concern, since excitatory/inhibitory (E/I) imbalance may act as an underlying mechanism of neurodevelopmental disorders (NDDs) (Lee et al., 2017; Sohal and Rubenstein, 2019). However, it should be noted that the neurotoxic effects of early, multiple anesthetic exposures have only been evaluated in normal mice (common inbred strains), but not in animal models of NDDs. Given the overlapping synaptic changes (E/I imbalance) observed in anesthesia-exposed animals and animal models of NDDs, it is possible that individuals with NDDs are more vulnerable to the neurotoxic effects of general anesthesia. Thus, unlike the overall population, where repeated anesthetic exposures may induce subtle changes, it is possible that patients with pre-existing cognitive impairments because of an E/I imbalance are at a higher risk due to the synergic effects of multiple anesthetic exposures.

BTBR T + Itpr3tf/J mice (hereinafter, BTBR mice) are an inbred strain that displays impaired sociability and repetitive behavior—the two core symptoms of autism spectrum disorder (ASD) (Meyza and Blanchard, 2017). Previous studies suggest that E/I imbalance, mitochondrial dysfunction, and changes in BDNF (brain-derived neurotrophic factor) expression are important mechanisms underlying autistic behavior in BTBR mice (Scattoni et al., 2013; Han et al., 2014; Cristiano et al., 2018; Ahn et al., 2020). Interestingly, exposures to anesthetics not only affect synaptic transmission, but also affect mitochondrial function and BDNF signaling (Lu et al., 2006; Chung et al., 2017a; Ju et al., 2019, 2020b; Woodward et al., 2019). Based on the overlapping changes seen in BTBR mice, we hypothesized that young BTBR mice could be significantly affected by sevoflurane, an inhalation agent widely used in pediatric patients. BTBR mice were exposed to sevoflurane at postnatal day 16, 17 (PND16/17), and further evaluated for possible changes in E/I imbalance, mitochondrial function, and BDNF signaling. Long-term changes in autistic behaviors were also evaluated in adult BTBR mice.

## Materials and Methods

### Animals

All procedures were approved by Chungnam National University Hospital, Daejeon, South Korea (CNUH-017-P0016). BTBR mice (Jackson laboratory, Bar Harbor, ME, United States) and C57BL/6J mice (RRID: MGI:5659186, Damul Science, Daejeon, South Korea) were housed in a controlled environment (temperature, 22°C; humidity, 40%) on a 12-h reverse light/dark cycle and allowed free access to food and water. Mice were weaned at PND21 and housed in groups of 3–5 per cage. All studies were performed in male mice, as the prevalence of autism is much higher in males.

### Anesthetic Exposures

BTBR mice were exposed to sevoflurane at PND16/17 as previously described (Ju et al., 2020a). Mice were randomly divided into four groups: a naïve C57BL/6J (B6-control) group, a B6-sevoflurane group, a BTBR-control group, and a BTBR-sevoflurane group. Mice in the B6/BTBR-sevoflurane group exposed to three cycles of 2.5% sevoflurane (Ilsung, Seoul, South Korea), delivered at a rate of 5 L/min at 36°C [inspired oxygen fraction (FiO_2_), 0.4]. Sevoflurane was administered for 1 h at each cycle, and there was a 2-h interval between cycles. BTBR-control and B6-control mice were exposed to the same constant fresh of gas but without sevoflurane. Sevoflurane and carbon dioxide (CO_2_) levels were monitored continuously (Dräger Vamos 2; Draegerwerk AG & Co., Germany).

### Electrophysiology

Whole-cell patch-clamp recordings of layer II and III pyramidal neurons in the medial prefrontal cortex (mPFC) were performed 5 days after sevoflurane exposures, as previous described (Han et al., 2014; Chung et al., 2017a). Coronal sections of the mPFC (300 μm) were cut in ice-cold, continuously aerated (95% O_2_/5% CO_2_) dissection buffer (sucrose-based) using a VT1200S vibratome (Leica, Switzerland). For recovery, the slices were transferred to chamber filled with aCSF warmed to 32°C for 30 min. Glass capillaries were filled with an internal solution whose composition depended on the experiment. To avoid obtaining too much data from a single slice or animal, the number of whole-cell recordings was limited to ≦2 cells per slice (containing one side of mPFC) and ≦5 cells per animal. For spontaneous inhibitory postsynaptic currents (sIPSCs) recordings, the solution consisted of 115 mM CsCl, 10 mM tetraethylammonium chloride, 8 mM NaCl, 10 mM HEPES, 5 mM QX-314-Cl, 4 mM Mg-ATP, 0.3 mM Na-guanosine triphosphate, and 10 mM EGTA. For spontaneous excitatory postsynaptic current (sEPSC) recordings, the solution consisted of 117 mM CsMeSO4, 10 mM tetraethylammonium chloride, 8 mM NaCl, 10 mM HEPES, 5 mM QX-314-Cl, 4 mM Mg-ATP, 0.3 mM Na-guanosine triphosphate, and 10 mM EGTA. Whole-cell voltage recordings were acquired at a holding potential of −70 mV in the presence of 50 μM picrotoxin (for sEPSCs) or 10 μM NBQX and 50 μM D-AP5 (for sIPSCs). Neurons were visually identified (BX50WI; Olympus, Japan), and recordings were performed using a MultiClamp 700B amplifier (Molecular Devices, United States). Data were acquired with Clampex 11.0.3 (Molecular Devices) and analyzed using Clampfit 11.0.3 (Molecular Devices).

### Mitochondrial Oxygen Consumption Rate

Mitochondria were isolated from the cerebral cortex 5 days after sevoflurane exposures, as previously described (Lee et al., 2021). Mitochondrial fraction was acquired from the cerebral cortex, and seeded in an XF-24 plate (Seahorse Bioscience, United States). Plates were centrifuged (2,000 × *g*, 20 min, 4°C) in a swinging bucket microplate adaptor (Eppendorf, Germany), after which 450 μl of mitochondrial assay buffer containing substrate (10 mM succinate plus 2 μM rotenone) was added to each well. The XF-24 plate was then placed in a CO_2_-free incubator and maintained at 37°C for 8–10 min, then loaded into the Seahorse XF-24 extracellular flux analyzer for OCR measurements.

### Western Blotting

Western blot was performed as previously described (Ju et al., 2020a). Protein samples of the cerebral cortex were obtained 5 days after sevoflurane exposures. The indicated primary antibodies against the following proteins were used: BDNF (Abcam, United Kingdom, CAT#ab226843), tropomyosin receptor kinase B (TrkB; Abcam, CAT#ab18987), phosphorylated TrkB (Sigma-Aldrich, Germany, CAT# ABN1381), OXPHOS (Abcam, CAT#ab110413), and β-actin (Santa Cruz Biotechnology, United States, CAT# sc-8432). Specific antibody-labeled proteins were detected using an enhanced chemiluminescence system (WEST-ZOL plus; intROn BioTechnology, South Korea).

### Behavioral Tests

All behavioral tests were performed blindly in adult mice (8–10 weeks) and video recorded. Time spent in each chamber in the three-chamber test was automatically measured using a tracking software (Ethovision XT; Noldus Information Technology, Netherlands). Grooming behavior was manually analyzed by a blinded observer.

### Three-Chamber Test

A three-chamber apparatus (60 cm width × 40 cm height × 22 cm depth) containing a cage in each side chamber was used to measure sociability. The experiment was composed of three 10-min sessions. In the first session, mice were restricted to the center chamber; in the second session, mice were allowed free access to explore all three chambers. After these two habituation sections, a novel object, consisting of a blue cube measuring 3.0 × 3.0 × 4.0 cm or a novel stranger mouse (age-matched male mouse of the same strain) was placed inside each side chamber. The subject mouse was allowed to freely explore the apparatus for 10 min, and the time spent sniffing the cages in each side chamber (containing a stranger mouse or novel object) was measured. The positions of the object and stranger were alternated each time to prevent side preference. Preference index (PI) was calculated using the time spent in each chamber (Mo, time spent in the mouse chamber; Ob, time spent in the object chamber): PI (%) = (Mo – Ob)/(Mo + Ob) × 100.

### Grooming Test

A home cage without any bedding was used for measuring grooming behavior (Chung et al., 2015). Mice were placed in the center of the cage for 20 min. Grooming was measured manually for 10 min after a 10-min adaptive phase. Cages were changed for each experiment.

### Statistical Analysis

Sample size was based on previous studies (Chung et al., 2017a; Ju et al., 2020a). Data were analyzed using the R statistical software package (4.0.3; R Core Team, Austria). All continuous variables were tested for conditions of normality and homogeneity of variance. When both conditions were met, a one-way analysis of variance (ANOVA) with *post hoc* Tukey’s HSD test was performed; when homogeneity of variance was unmet, Welch’s ANOVA with *post hoc* Tukey’s HSD test was performed; and when normality was unmet, the Kruskal–Wallis test with *post hoc* Dunn’s test was performed. Statistics are presented as Supplementary Data.

## Results

### Sevoflurane Exposures Improves E/I Balance in Late Postnatal BTBR Mice

**FIGURE 1:**
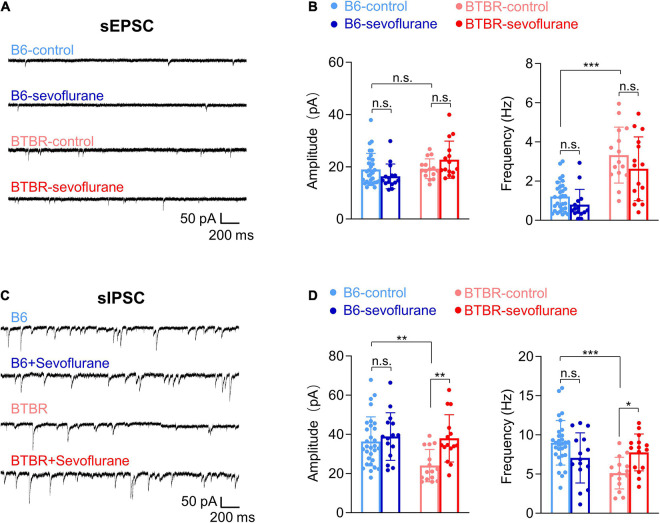
Sevoflurane exposures induce excitatory/inhibitory imbalance in late postnatal BTBR mice. Postsynaptic currents were measured in pyramidal neurons (mPFC layer II, III) 5 days after mice received three episodes of sevoflurane exposure at 2-h intervals. **(A)** Examples of sEPSCs. **(B)** Quantification of sEPSC amplitude (pA) and frequency (Hz) (B6-control, *n* = 30 cells from seven mice; B6-sevoflurane, *n* = 15 cells from three mice; BTBR-control, *n* = 15 cells from four mice; BTBR-sevoflurane, *n* = 15 cells from three mice). **(C)** Examples of sIPSCs. **(D)** Quantification of sIPSC amplitude (pA) and frequency (Hz) (B6-control, *n* = 30 cells from seven mice; B6-sevoflurane, *n* = 15 cells from three mice; BTBR-control, *n* = 15 cells from four mice; BTBR-sevoflurane, *n* = 15 cells from three mice). Values are presented as means ± SD (*p < 0.05, **p < 0.01, ***p < 0.001, n.s., not significant).

E/I imbalance during neurodevelopment is a major mechanism underlying autism and a reported feature in BTBR mice (Han et al., 2014). As we previously reported that sevoflurane exposures induce long-lasting E/I imbalance (Ju et al., 2020a), changes in E/I balance were evaluated by measuring spontaneous synaptic transmission in pyramidal neurons of the mPFC (layer II, III), a brain region involved in autism behavior (Chung et al., 2015; Kim et al., 2016). Excitatory synaptic transmission was increased in BTBR mice compared with B6 mice, as evidenced by the significantly increased frequency (but not amplitude) of sEPSCs (Figures 1A,B; Welch’s ANOVA with *post hoc* Tukey’s HSD test, *p* < 0.001). Such differences in excitatory synaptic transmission were not affected by early sevoflurane exposures (Figures 1A,B). We also discovered that inhibitory synaptic transmission was decreased in BTBR mice, as shown by a significant decrease in sIPSC amplitude and frequency compared with B6 mice (Figures 1C,D) (Amplitude, Kruskal–Wallis test with *post hoc* Dunn’s test, *p* = 0.004; Frequency, ANOVA with *post hoc* Tukey’s HSD test, *p* < 0.001). Interestingly, early sevoflurane exposures significantly increased both amplitude and frequency of sIPSCs in BTBR mice (Figures 1C,D; Amplitude, Kruskal–Wallis test with *post hoc* Dunn’s test, *p* = 0.005; Frequency, ANOVA with *post hoc* Tukey’s HSD test, *p* = 0.043). Collectively, these results suggest that early sevoflurane exposures improve pre-existing E/I imbalances in BTBR mice.

### Sevoflurane Exposures Improve Mitochondrial Respiration in Late Postnatal BTBR Mice

**FIGURE 2:**
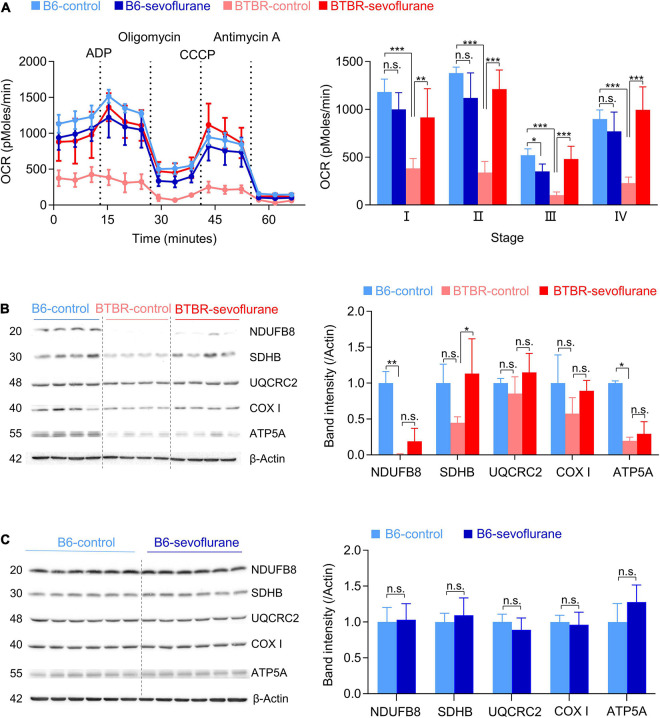
Sevoflurane exposures induce a long-lasting increase in mitochondrial respiration in late postnatal BTBR mice. OCR and expression of mitochondrial complex subunits were measured from the cerebral cortex 5 days after sevoflurane exposures. **(A)** OCR measurements were obtained in five stages: I, basal stage OCR; II, OCR during ATP production, measured by adding ADP; III, OCR from protein leakage, measured by adding oligomycin; IV, uncoupled respiration, measured by adding CCCP; V, non-mitochondrial respiration, measured by adding antimycin A. Dotted lines indicate the time at which specific drugs were added. Average OCR (three measurements in every stage for each brain sample) was compared between groups. (B6-control, *n* = 5 mice; B6-sevoflurane, *n* = 5 mice; BTBR-control, *n* = 4 mice; BTBR-sevoflurane, *n* = 5 mice). **(B,C)** Western blot results of the five mitochondrial complex subunits. Numbers on the left of the images indicate protein size (kDa). **(B)** Protein expression levels were measured in B6-control, BTBR-control, and BTBR-sevoflurane mice (*n* = 4 per group). **(C)** Protein expression levels were measured in B6-control and B6-sevoflurane mice (*n* = 6 per group). Values are presented as mean ± SD (*p < 0.05, **p < 0.01, ***p < 0.001, n.s., not significant).

Previous studies suggest multiple mechanisms for the autistic behavior in BTBR mice, including suboptimal mitochondrial function (Ahn et al., 2020). Since sevoflurane can also affect mitochondrial function (Chung et al., 2017a; Ju et al., 2020b), we next evaluated the effects of early sevoflurane exposures on mitochondrial respiration (OCR). Consistent with previous studies, mitochondrial OCR was reduced in BTBR-control mice compared with that in B6-control mice (Figure 2A; one-way ANOVA with *post hoc* Tukey’s HSD test, *p* < 0.001). Interestingly, early sevoflurane exposures significantly increased mitochondrial respiration in BTBR mice (BTBR-control vs BTBR-Sevoflurane) (Figure 2A; one-way ANOVA with *post hoc* Tukey’s HSD test; Stage I, *p* = 0.005; II, *p* < 0.001; III, *p* < 0.001; IV, *p* < 0.001). We also measured expression levels of mitochondrial oxidative phosphorylation (OXPHOS) complex subunits. The expression of NDUFB8 [NADH dehydrogenase (ubiquinone) 1 beta subcomplex subunit 8, complex I] and ATP5A (mitochondrial α-F_1_-ATP synthase subunit, complex V) were significantly decreased in BTBR mice compared with B6 mice (Figure 2B; NDUFB8, Kruskal–Wallis test, *p* = 0.005; ATP5A, Kruskal–Wallis test, *p* = 0.03). However, only the expression of SDHB (succinate dehydrogenase (ubiquinone) iron-sulfur subunit, complex II) was increased in BTBR mice after early multiple exposures to sevoflurane (Figure 2B; One-way ANOVA with *post hoc* Tukey’s HSD test, *p* = 0.036). Unlike BTBR mice, sevoflurane exposures did not affect expression of mitochondrial oxidative phosphorylation (OXPHOS) complex subunits in B6 mice (Figure 2C).

### Sevoflurane Exposures Increase BDNF/Tropomyosin Receptor Kinase B Signaling in Late Postnatal BTBR Mice

**FIGURE 3:**
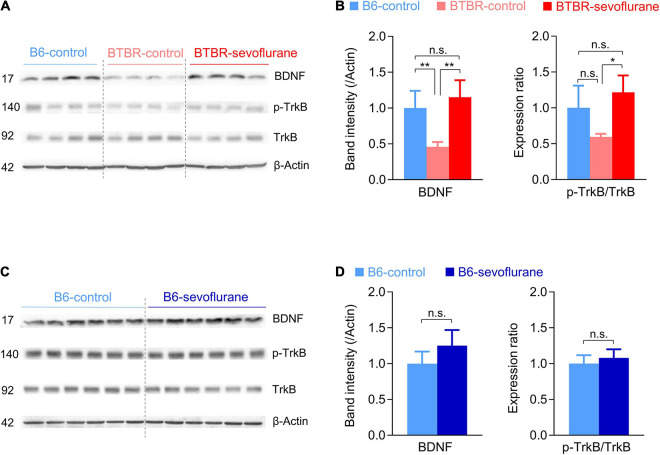
Sevoflurane exposures increase BDNF/TrkB signaling in late postnatal BTBR mice. **(A–D)** Expression levels of BDNF and TrkB were measured in cerebral cortex samples obtained 5 days after sevoflurane exposures. **(A,B)** Western blot images and quantification of BDNF/TrkB signaling expression in B6-control, BTBR-control, and BTBR-sevoflurane mice (*n* = 4 mice for all groups). **(C,D)** Western blot images and quantification of BDNF/TrkB signaling expression in B6-control and B6-sevoflurane mice (*n* = 6 per group). Numbers on the left indicate protein size (kDa). Values are presented as mean ± SD (*p < 0.05, **p < 0.01, n.s., not significant).

While our results suggest that early sevoflurane exposures correct both E/I imbalance and mitochondrial dysfunction, it raises the question of a common upstream signaling pathway. In this context, the BDNF/TrkB signaling pathway may be involved, since it not only regulates both mitochondrial function and synaptic transmission (Markham et al., 2004, 2012; Gottmann et al., 2009), but is also reduced in BTBR mice (Scattoni et al., 2013; Cristiano et al., 2018; Rhine et al., 2019). Consistent with previous studies, BDNF expression was significantly decreased in the cerebral cortex of BTBR-control mice compared with B6-control mice (Figures 3A,B; One-way ANOVA with *post hoc* Tukey’s HSD test, *p* = 0.01). Notably, early sevoflurane exposures in BTBR mice significantly increased this low basal BDNF expression, restoring BDNF to levels similar to those in B6-control mice (Figures 3A,B). We next measured TrkB phosphorylation, the main receptor of BDNF. Whereas phospho-TrkB/total-TrkB ratios were not significantly different between B6-control and BTBR-control mice, early sevoflurane exposures also increased the phospho-TrkB/total-TrkB ratio in BTBR mice (Figures 3A,B; Welch ANOVA with *post hoc* Tukey’s HSD test, *p* = 0.02). Similar to the mitochondrial results, sevoflurane exposures did not affect BDNF signaling in B6 mice (Figures 3C,D).

### Sevoflurane Exposures During Neurodevelopment Restore Sociability and Reduce Repetitive Behavior in Adult BTBR Male Mice

**FIGURE 4:**
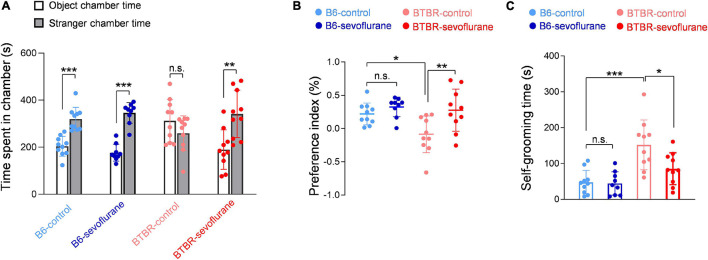
Autistic behavior in BTBR mice is reduced by early sevoflurane exposures in BTBR mice. **(A,B)** Sociability was evaluated using the three-chamber test by measuring the time spent in each side chamber. **(A)** Quantification of the time spent in each side chamber (B6-control, *n* = 10 mice; B6-sevoflurane, *n* = 9 mice; BTBR-control, *n* = 10 mice; BTBR-sevoflurane, *n* = 10 mice). **(B)** Quantification of the preference index (B6-control, *n* = 10 mice; B6-sevoflurane, *n* = 9 mice; BTBR-control, *n* = 10 mice; BTBR-sevoflurane, *n* = 10 mice). **(C)** Repetitive behavior was evaluated by measuring the duration of self-grooming behavior. (B6-control, *n* = 10 mice; B6-sevoflurane, *n* = 9 mice; BTBR-control, *n* = 10 mice; BTBR-sevoflurane, *n* = 10 mice). Values are presented as means ± SD (*p < 0.05, **p < 0.01, ***p < 0.001, n.s., not significant).

Since our results suggest that early sevoflurane exposures correct pathophysiological mechanisms involved with autistic behavior in BTBR mice, we next investigated for possible changes in autistic behavior. We first tested sociability using the three-chamber test. We initially discovered that both B6 and BTBR mice spent a significant amount of time sniffing the cage containing the stranger mouse (Supplementary Figure 1), an unexpected result given that impaired sociability is a well-established feature of BTBR mice. Previous studies have also shown that BTBR mice may express significant sociability under certain experimental conditions (Bales et al., 2014; Segal-Gavish et al., 2016). A possible explanation for our initial, apparently anomalous, results may be differences in the three-chamber test protocol. We initially used a relatively small three-chamber apparatus (Chung et al., 2015), and used aged-matched B6 mice as a stranger mouse regardless of the strain of the subject mouse. However, most previous studies performed the three-chamber test using a much larger apparatus (Moy et al., 2007), and several studies suggest that the strain of the stranger mouse may have significant effects (Ryan et al., 2019). Thus, we repeated the experiments using a larger three-chamber apparatus and changed the stranger mice to the same strain as the subject mouse. Under these conditions, B6-control mice continued to prefer sniffing the cage containing the stranger mouse (Figure 4A; One-way ANOVA, *p* < 0.001), whereas BTBR-control mice showed no significant difference in the time spent sniffing the cage containing the stranger mouse and that containing an inanimate object (Figure 4A). However, BTBR-sevoflurane mice displayed normal sociability by spending significantly more time in the chamber containing the stranger mouse (Figure 4A; One-way ANOVA, *p* = 0.002). The preference index in the three-chamber test was also increased after early sevoflurane exposures in BTBR mice (Figure 4B; One-way ANOVA with *post hoc* Tukey’s HSD test, *p* = 0.009), further suggesting that sevoflurane exposures during neurodevelopment may improve sociability in BTBR mice. Self-grooming is often increased in animal models of ASD and has been suggested to represent restricted, repetitive behavior. Similar to previous results, grooming behavior was significantly increased in BTBR-control mice compared with B6-control mice (Figure 4C; One-way ANOVA with *post hoc* Tukey’s HSD test, *p* < 0.001). Interestingly, this increased grooming behavior in BTBR mice was also reduced by early sevoflurane exposures (Figure 4C; One-way ANOVA with *post hoc* Tukey’s HSD test, *p* = 0.018). Our behavioral results thus suggest that early sevoflurane exposures may reduce autistic behavior by correcting the pathophysiological mechanisms involved in BTBR mice.

## Discussion

Our group recently reported that, unlike a single exposure (Chung et al., 2017a), multiple sevoflurane exposures administered over a short interval induce long-term neurological changes (Ju et al., 2020a). In the present study, we further measured long-term changes after early anesthetic exposures in BTBR mice, a widely studied autism mouse model, in order to determine whether repeated exposure to anesthetics during neurodevelopment has a different effect in individuals with preexisting neurological changes. Most unexpectedly, our results showed that early sevoflurane exposures beneficially affected pathological changes suggested as important mechanisms for autistic behavior in BTBR mice, rescuing the E/I imbalance, increasing mitochondrial respiration, and activating the BDNF/TrkB pathway. We also discovered that early anesthetic exposures do not aggravate autistic behavior, but may actually improve sociability and reduce self-grooming.

E/I imbalance is closely associated with NDDs (Lee et al., 2017; Sohal and Rubenstein, 2019), and reduced GABAergic transmission has been reported in autism patients and autism model mice (Fatemi et al., 2009; Oblak et al., 2009; Han et al., 2012, 2014). A previous study also reported reduced inhibitory synaptic transmission and enhanced excitatory synaptic transmission in BTBR hippocampal pyramidal neurons (PND 21-25) (Han et al., 2014). These studies showed that pharmacologically enhancing GABAergic transmission can ameliorate behavioral deficits in animal models of autism, including BTBR mice (Han et al., 2014; Silverman et al., 2015; Yoshimura et al., 2017; Rhine et al., 2019). Our results suggest an E/I imbalance in pyramidal neurons of the mPFC similar to that previously reported in the hippocampus (Han et al., 2014), as we found an increase in sEPSC frequency and a decrease in sIPSC frequency. Importantly, early sevoflurane exposures increased inhibitory synaptic transmission in BTBR mice, measured 5 days after anesthetic exposure. By improving E/I balance, such changes may be involved in decreasing autistic behavior in BTBR mice. Notably, the consequences of sevoflurane exposures in BTBR male mice are not consistent with our recent study performed in male B6 mice, in which short-interval sevoflurane exposures did not affect inhibitory synaptic transmission (Ju et al., 2020a). However, it is difficult to directly compare these results due to differences in brain region (hippocampus vs. mPFC) and the presence of tetrodotoxin (TTX) during synaptic transmission measurements. We previously measured changes in synaptic transmission after eliminating neuronal activity by adding TTX in the bath solution (miniature EPSC/IPSC) (Ju et al., 2020a). However, based on a previous BTBR study regarding E/I balance (Han et al., 2014), we evaluated changes in synaptic transmission in BTBR mice without TTX (sEPSC/IPSC). Spontaneous synaptic transmission is also affected by changes in network activity, which may be involved with the inconsistent changes in inhibitory synaptic transmission.

While our results are in line with previous studies suggesting that attenuating possible mechanisms of autism may reduce autistic behavior (Geschwind, 2008), it is somewhat unexpected that early anesthetic exposures improve multiple mechanisms involved with autistic behavior in BTBR mice. It is highly possible that sevoflurane-induced changes in synaptic transmission, mitochondrial respiration, and BDNF signaling are inter-related. For instance, studies have reported that BDNF/TrkB signaling affects mitochondria function by increasing mitochondrial respiration (Markham et al., 2004, 2012), suggesting that increased BDNF signaling after sevoflurane exposures is involved in the increase in mitochondrial OCR. Previous studies also suggest that BDNF regulates inhibitory synaptic transmission. However, the effects of BDNF on inhibitory synaptic transmission may depend on the brain region, cell type, and developmental stage (Gottmann et al., 2009). Whereas acute BDNF treatment reduces inhibitory synaptic transmission in hippocampus slices (Tanaka et al., 1997), it was reported to increase inhibitory synaptic transmission in freshly isolated cells from the visual cortex (Mizoguchi et al., 2003). The duration of BDNF activation is also an important factor, since studies have shown that BDNF acutely reduces but chronically increases inhibitory synaptic transmission (Gottmann et al., 2009). Although our results suggest that activation of BDNF signaling by sevoflurane exposures may increase inhibitory synaptic transmission in the mPFC, further studies focusing on other brain regions and ages are warranted.

Age is an important factor when studying anesthesia-induced neurotoxicity in young rodents. Although the majority of studies have reported widespread neuronal cell death in neonatal rodents (PND7) (Lee and Loepke, 2018), several studies in late postnatal mice (PND17) have reported increased dendritic spinogenesis and E/I imbalance (Chung et al., 2017a; Ju et al., 2019, 2020a,2020b). Thus, it is possible that the effects of sevoflurane exposures in BTBR mice may differ depending on the neurodevelopmental stage. Importantly, the neurodevelopment of PND7 mice may be comparable to a third-trimester human fetus (Workman et al., 2013; Charvet, 2020). Considering that the majority of clinical studies focus on infants and very young children, we exposed mice at PND16/17, an age that may be more comparable to a human infant (Workman et al., 2013; Charvet, 2020). However, further studies at PND7 are also necessary, as it is difficult to compare neurodevelopment between humans and mice.

Our study is not without limitations. First, it is possible that our results in BTBR mice may not be observed in other autism animal models. Although studies have shown that BTBR mice share important changes in relevant genes and proteins involved with autism (Daimon et al., 2015), additional studies in mice carrying mutations in specific genes involved in autism (ex, CHD8, Shank3) are necessary to recognize the impact of early anesthetic exposures in NDDs. The second limitation lies with the anesthetic protocol itself. BTBR mice received three sevoflurane exposures at 2-h intervals based on previous results (Ju et al., 2020a). While this protocol has the advantage of providing extended anesthesia exposure without causing significant physiological changes (Ju et al., 2020a) or general health (based on weight change, Supplementary Figure 2), it does not address the effects of a single anesthetic exposure or multiple exposures with longer intervals. Another important limitation of our study is that we did not distinguish subregions of the mPFC. Recent studies suggested that pyramidal neurons in the mPFC display layer-, subregion-specific differences in neurophysiologic properties (Song and Moyer, 2018). Although all electrophysiology studies were performed in an identical fashion by a single experimenter, such subregion-specific differences may have affected our results. The fourth limitation is that we have not established a causal relationship between the changes in BDNF signaling, mitochondrial function, synaptic transmission, and behavior. Although unlikely, it is possible that the changes in BDNF signaling, mitochondrial function, synaptic transmission, and behavior are independent.

In conclusion, multiple sevoflurane exposures correct potential pathophysiological mechanisms of autistic behavior in late postnatal BTBR mice. Not only do our results provide additional evidence of long-lasting neurological changes after multiple anesthetic exposures during neurodevelopment, they also suggest that the observed changes may affect individuals diagnosed with NDDs differently. Future studies focusing on diverse animal models of NDDs will help understand the significance of anesthetic exposures during neurodevelopment.

## Data Availability Statement

The original contributions presented in the study are included in the article/Supplementary Material, further inquiries can be directed to the corresponding author/s.

## Ethics Statement

The animal study was reviewed and approved by Chungnam National University Hospital, Daejeon, South Korea (CNUH-017-P0016).

## Author Contributions

JC and JP designed, developed, and executed the experiments and prepared the manuscript. BH analyzed the data. XJ, YL, and JA performed the experiments. YHK, YK, S-HY, and S-OH helped in the design and development of the experiments. WC and JH designed, developed, oversaw, and prepared the manuscript. All authors contributed to the article and approved the submitted version.

## Conflict of Interest

The authors declare that the research was conducted in the absence of any commercial or financial relationships that could be construed as a potential conflict of interest.

## Publisher’s Note

All claims expressed in this article are solely those of the authors and do not necessarily represent those of their affiliated organizations, or those of the publisher, the editors and the reviewers. Any product that may be evaluated in this article, or claim that may be made by its manufacturer, is not guaranteed or endorsed by the publisher.
