# Supplementary material for: General Anesthesia During Neurodevelopment Reduces Autistic Behavior in Adult BTBR Mice, a Murine Model of Autism
Source: Front Cell Neurosci. 2021 Nov 29;15:772047. doi: 10.3389/fncel.2021.772047 (PMC8667765; doi:10.3389/fncel.2021.772047)
Supplement: Supplementary file 1 [file Data_Sheet_1.PDF]

1. Supplementary Figures 1- 2

Suppl Fig 1

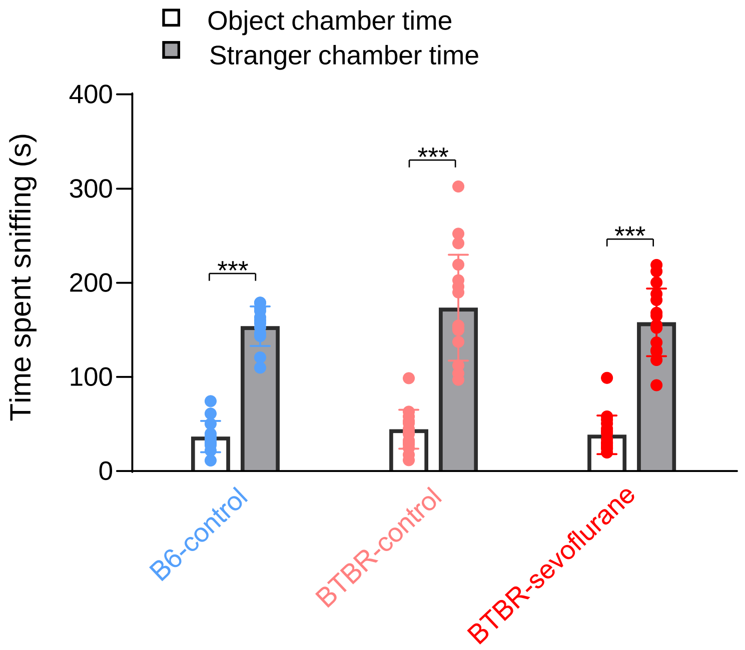

Suppl Fig 1. Both B6 and BTBR mice display a preference for the stranger mouse in the 3-chamber test when using a relatively small sized 3-chamber apparatus and unifying the stranger mouse with an aged-matched B6 mouse. Sociability was assessed in a relatively small three-chamber apparatus (40 cm width × 20 cm height × 26 cm depth [12-cm-wide center chamber, 14-cm-wide side chambers]). Age-matched B6 male mice were used as stranger mouse regardless of the subject mouse. Time spent sniffing cages in each chamber were measured to assess sociability. Mice in all groups spent significantly more time sniffing the cage containing the stranger mouse compared to the cage containing a novel object (n = 13 for B6-control, n = 17 for BTBR-control, n = 16 for BTBR-sevoflurane). Values are presented as means ± SD. Welch ANOVA: B6-control, $p<0.001$; BTBR-control, $p<0.001$;BTBR-sevoflurane, $p<0.001$ (***p <0.001).

Suppl Fig 2

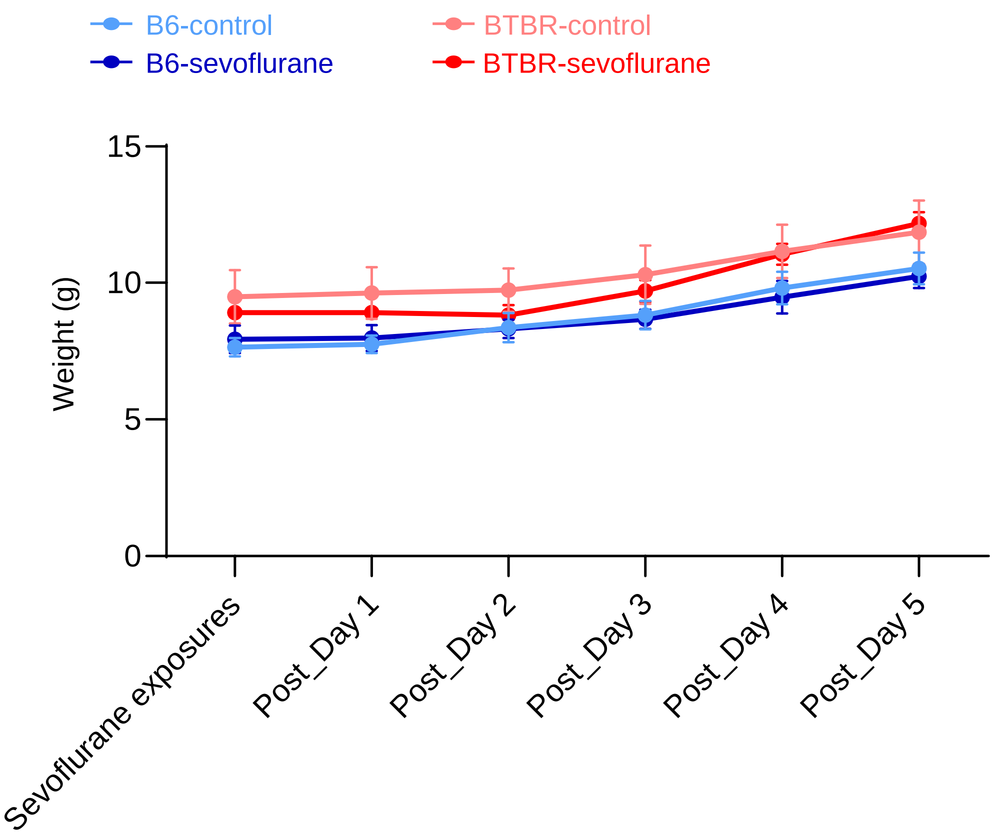

Suppl Fig 2. Body weight is not affected by sevoflurane exposures at postnatal day 16,17

2. The results of statistical analysis
(Figure 1-4 and Supplementary Figure 1)

2.1 The results of statistical analysis

Figure 1

Fig 1 B. sEPSC amplitude Data analysis using R

Boohwi Hong

Package install

Data import

Data structure

```
str(d1)
```

```
## 'data.frame':    75 obs. of  3 variables:
##  $ subject: int  1 2 3 4 5 6 7 8 9 10 ...
##  $ group  : chr  "B6_con" "B6_con" "B6_con" "B6_con" ...
##  $ ampl   : num  13.6 18.8 12.2 16.7 18.7 ...
```

Explorative data analysis with graphics

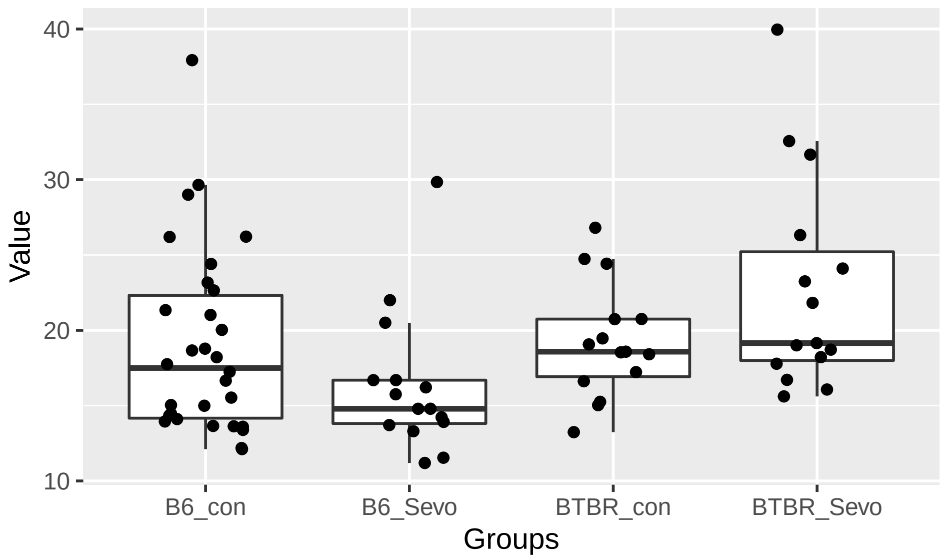

Easystat function developed by S. Park (available at https://rpubs.com/goodlebang)

Statistical Result

```
easystat(d1)
```

```
## 1. Normality assumption test by Shapiro_Wilk test is
##  p = 0.000
##  Normality assumption was rejected
## 2. The result of Kruskall_Wallis test:
##  p = 0.011
##  A statistically significant difference exist between groups
```

```
## Dunn (1964) Kruskal-Wallis multiple comparison
```

```
##   p-values adjusted with the Benjamini-Hochberg method.
```

```
##              Comparison          Z     P.unadj       P.adj
## 1     B6_con - B6_Sevo  1.5283584 0.126423584 0.189635376
## 2    B6_con - BTBR_con -0.8996033 0.368331375 0.368331375
## 3   B6_Sevo - BTBR_con -2.1026765 0.035494055 0.070988110
## 4   B6_con - BTBR_Sevo -2.1764597 0.029520903 0.088562710
## 5  B6_Sevo - BTBR_Sevo -3.2084666 0.001334448 0.008006689
## 6 BTBR_con - BTBR_Sevo -1.1057900 0.268817404 0.322580884
```

Fig 1 B. sEPSC frequency Data analysis using R

Boohwi Hong

Package install

Data import

Data structure

```
str(d1)
```

```
## 'data.frame':    75 obs. of  3 variables:
##  $ subject: int  1 2 3 4 5 6 7 8 9 10 ...
##  $ group  : chr  "B6_con" "B6_con" "B6_con" "B6_con" ...
##  $ freq   : num  1.617 2.025 0.392 0.475 2.15 ...
```

Explorative data analysis with graphics

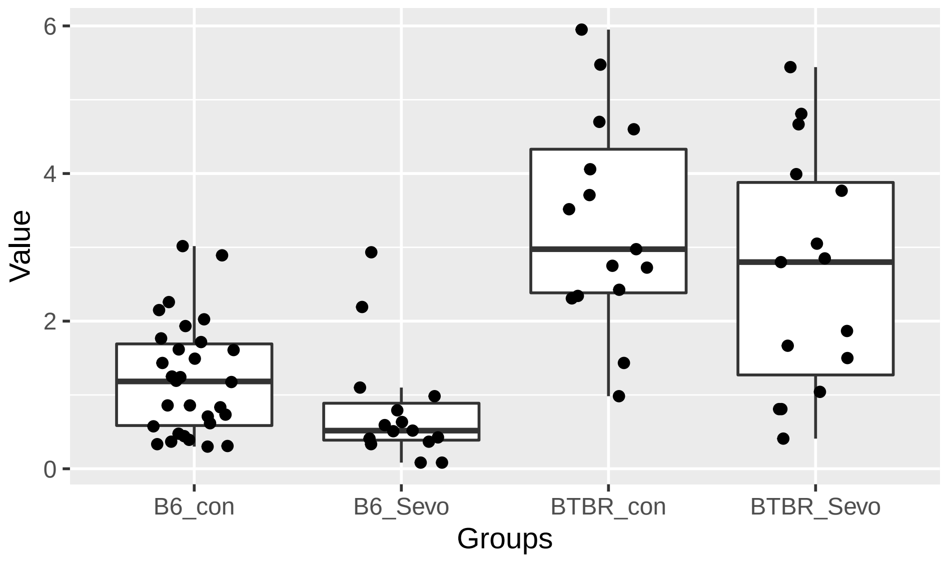

Easystat function developed by S. Park (available at https://rpubs.com/goodlebang)

Statistical Result

```r
easystat(d1)
```

```
## 1. Normality assumption test by Shapiro_Wilk test is
##  p = 0.061
##  Normality assumption was not rejected
## 2. Equal variance test by Bartlett test is
##  p = 0.001
##  Equal variance assumption was rejected
## 3. The result of Welch ANOVA is
##  p = 0.000
##  A statistically significant difference exist between groups
```

```
##   Tukey multiple comparisons of means
##     95% family-wise confidence level
##
## Fit: aov(formula = d1[, 3] ~ d1[, 2])
##
## $`d1[, 2]`
##                         diff        lwr       upr     p adj
## B6_Sevo-B6_con     -0.4222223 -1.3637037 0.5192591 0.6414438
## BTBR_con-B6_con     2.1111111  1.1696297 3.0525925 0.0000007
## BTBR_Sevo-B6_con    1.4127778  0.4712964 2.3542592 0.0010331
## BTBR_con-B6_Sevo    2.5333334  1.4462043 3.6204625 0.0000003
## BTBR_Sevo-B6_Sevo   1.8350001  0.7478710 2.9221292 0.0001858
## BTBR_Sevo-BTBR_con -0.6983333 -1.7854624 0.3887958 0.3365166
```

Fig 1 D. sIPSC amplitude Data analysis using R

Boohwi Hong

Package install

Data import

Data structure

```
str(d1)
```

```
## 'data.frame':    75 obs. of  3 variables:
##  $ subject: int  1 2 3 4 5 6 7 8 9 10 ...
##  $ group  : chr  "B6_con" "B6_con" "B6_con" "B6_con" ...
##  $ amp    : num  31.9 23 58.1 36.5 36.9 ...
```

Explorative data analysis with graphics

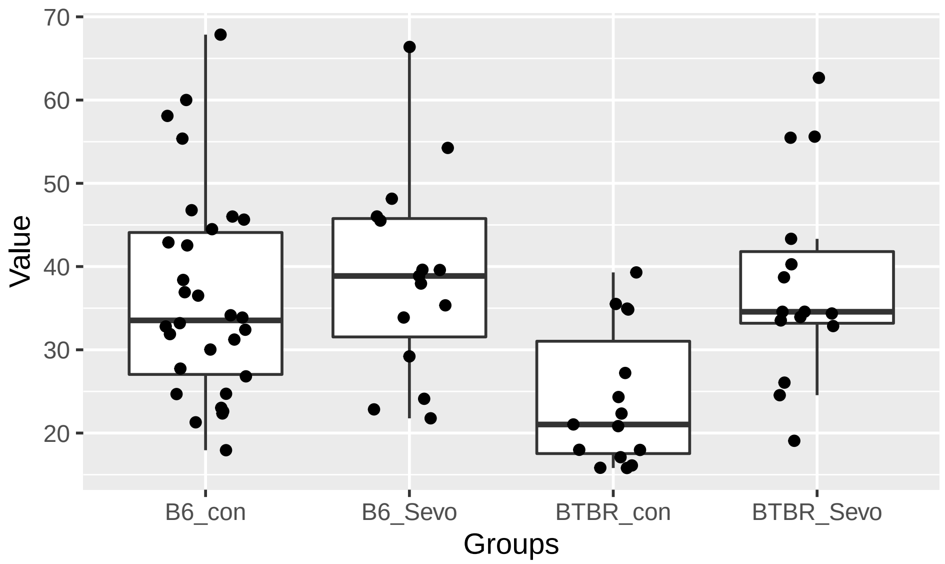

Easystat function developed by S. Park (available at https://rpubs.com/goodlebang)

Statistical Result

```
easystat(d1)
```

```
## 1. Normality assumption test by Shapiro_Wilk test is
##  p = 0.011
##  Normality assumption was rejected
## 2. The result of Kruskall_Wallis test:
##  p = 0.002
##  A statistically significant difference exist between groups
```

```
## Dunn (1964) Kruskal-Wallis multiple comparison
```

```
##   p-values adjusted with the Benjamini-Hochberg method.
```

```
##             Comparison          Z      P.unadj       P.adj
## 1     B6_con - B6_Sevo -0.8899175 0.3735101685 0.560265253
## 2    B6_con - BTBR_con  3.0953653 0.0019657061 0.003931412
## 3   B6_Sevo - BTBR_con  3.4513562 0.0005577769 0.003346661
## 4   B6_con - BTBR_Sevo -0.5126699 0.6081822599 0.729818712
## 5  B6_Sevo - BTBR_Sevo  0.3267060 0.7438902251 0.743890225
## 6 BTBR_con - BTBR_Sevo -3.1246502 0.0017801664 0.005340499
```

Fig 1 D. sIPSC frequency Data analysis using R

Boohwi Hong

Package install

Data import

Data structure

```
str(d1)
```

```
## 'data.frame':    75 obs. of  3 variables:
##  $ subject: int  1 2 3 4 5 6 7 8 9 10 ...
##  $ group  : chr  "B6_con" "B6_con" "B6_con" "B6_con" ...
##  $ freq   : num  4.76 7.35 15.98 3.25 7.12 ...
```

Explorative data analysis with graphics

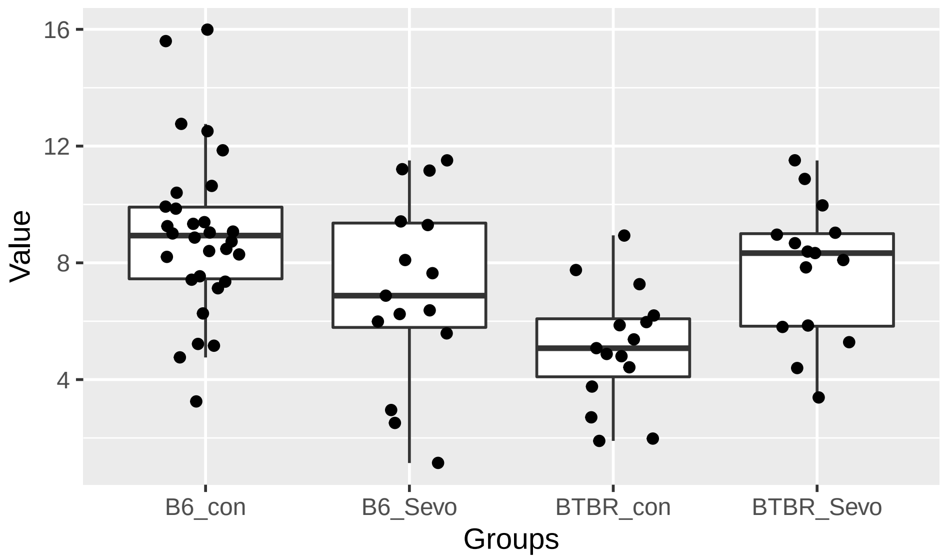

Easystat function developed by S. Park (available at https://rpubs.com/goodlebang)

Statistical Result

```
easystat(d1)
```

```
## 1. Normality assumption test by Shapiro_Wilk test is
##  p = 0.569
##  Normality assumption was not rejected
## 2. Equal variance test by Bartlett test is
##  p = 0.336
##  Equal variance assumption was not rejected
## 3. The result of anova is
##  p = 0.000
##  A statistically significant difference exist between groups
```

```
##   Tukey multiple comparisons of means
##     95% family-wise confidence level
##
## Fit: aov(formula = d1[, 3] ~ d1[, 2], data = d1)
##
## $`d1[, 2]`
##                         diff         lwr        upr     p adj
## B6_Sevo-B6_con     -1.9211111 -4.15393822  0.3117160 0.1164625
## BTBR_con-B6_con    -3.8632221 -6.09604918 -1.6303949 0.0001242
## BTBR_Sevo-B6_con   -1.2288890 -3.46171609  1.0039382 0.4741457
## BTBR_con-B6_Sevo   -1.9421110 -4.52035764  0.6361357 0.2045496
## BTBR_Sevo-B6_Sevo   0.6922221 -1.88602455  3.2704688 0.8942510
## BTBR_Sevo-BTBR_con  2.6343331  0.05608641  5.2125798 0.0433660
```

2.2 The results of statistical analysis

Figure 2

Fig 2 A. stage I Data analysis using R

Boohwi Hong

Package install

Data import

Data structure

```
str(d1)
```

```
## 'data.frame':    19 obs. of  3 variables:
##  $ subject: int  1 2 3 4 5 6 7 8 9 10 ...
##  $ group  : chr  "B6_con" "B6_con" "B6_con" "B6_con" ...
##  $ stage.I: num  1408 1100 1157 1180 1063 ...
```

Explorative data analysis with graphics

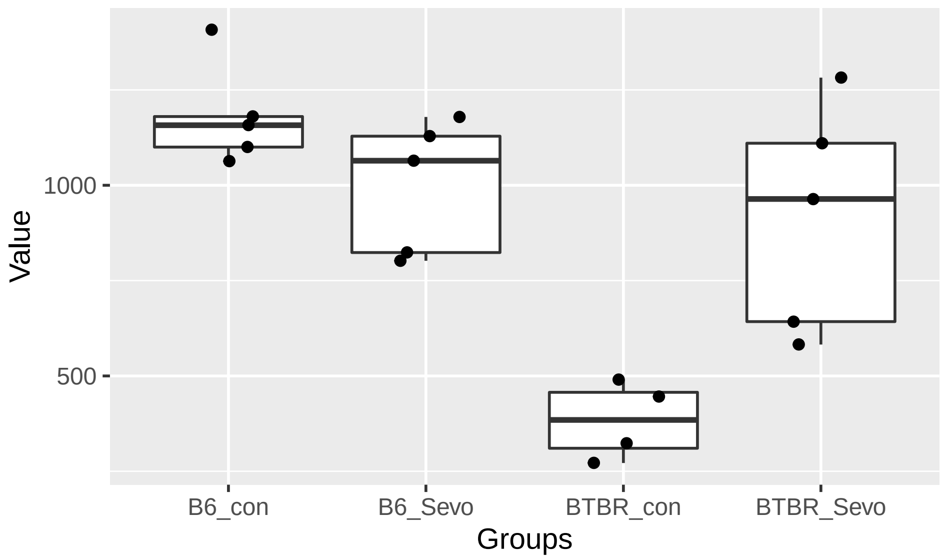

Easystat function developed by S. Park (available at https://rpubs.com/goodlebang)

Statistical Result

```
easystat(d1)
```

```
## 1. Normality assumption test by Shapiro_Wilk test is
##  p = 1
##  Normality assumption was not rejected
## 2. Equal variance test by Bartlett test is
##  p = 0.241
##  Equal variance assumption was not rejected
## 3. The result of anova is
##  p = 0.000
##  A statistically significant difference exist between groups
```

```
##   Tukey multiple comparisons of means
##     95% family-wise confidence level
##
## Fit: aov(formula = d1[, 3] ~ d1[, 2], data = d1)
##
## $`d1[, 2]`
##                          diff        lwr        upr     p adj
## B6_Sevo-B6_con     -182.29054  -542.9916  178.41050 0.4859636
## BTBR_con-B6_con    -799.05402 -1181.6353 -416.47280 0.0001242
## BTBR_Sevo-B6_con   -265.59858  -626.2996   95.10246 0.1907749
## BTBR_con-B6_Sevo   -616.76348  -999.3447 -234.18226 0.0016077
## BTBR_Sevo-B6_Sevo   -83.30804  -444.0091  277.39300 0.9083378
## BTBR_Sevo-BTBR_con  533.45544   150.8742  916.03667 0.0054829
```

Fig 2 A. stage II Data analysis using R

Boohwi Hong

Package install

Data import

Data structure

```
str(d1)
```

```
## 'data.frame':    19 obs. of  3 variables:
##  $ subject : int  1 2 3 4 5 6 7 8 9 10 ...
##  $ group   : chr  "B6_con" "B6_con" "B6_con" "B6_con" ...
##  $ stage.II: num  1426 1280 1417 1419 1356 ...
```

Explorative data analysis with graphics

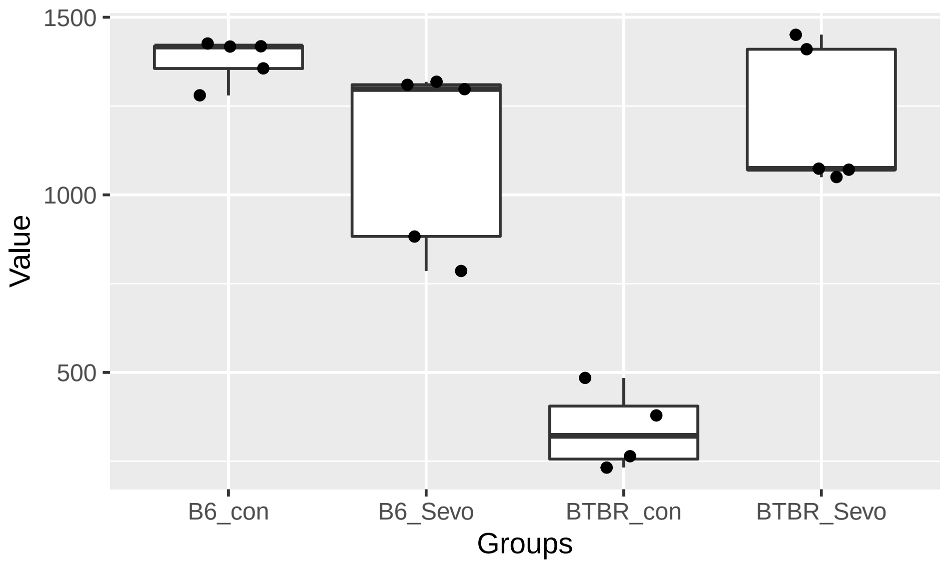

Easystat function developed by S. Park (available at https://rpubs.com/goodlebang)

Statistical Result

```
easystat(d1)
```

```
## 1. Normality assumption test by Shapiro_Wilk test is
##  p = 0.376
##  Normality assumption was not rejected
## 2. Equal variance test by Bartlett test is
##  p = 0.083
##  Equal variance assumption was not rejected
## 3. The result of anova is
##  p = 0.000
##  A statistically significant difference exist between groups
```

```
##   Tukey multiple comparisons of means
##     95% family-wise confidence level
##
## Fit: aov(formula = d1[, 3] ~ d1[, 2], data = d1)
##
## $`d1[, 2]`
##                          diff        lwr        upr     p adj
## B6_Sevo-B6_con      -260.56292  -590.7476   69.62174 0.1484804
## BTBR_con-B6_con    -1039.68607 -1389.8998 -689.47236 0.0000020
## BTBR_Sevo-B6_con    -168.54560  -498.7303  161.63906 0.4777205
## BTBR_con-B6_Sevo    -779.12316 -1129.3369 -428.90944 0.0000623
## BTBR_Sevo-B6_Sevo     92.01732  -238.1673  422.20198 0.8518835
## BTBR_Sevo-BTBR_con   871.14048   520.9268 1221.35419 0.0000174
```

Fig 2 A. stage III Data analysis using R

Boohwi Hong

Package install

Data import

Data structure

```
str(d1)
```

```
## 'data.frame':    19 obs. of  3 variables:
##  $ subject  : int  1 2 3 4 5 6 7 8 9 10 ...
##  $ group    : chr  "B6_con" "B6_con" "B6_con" "B6_con" ...
##  $ stage.III: num  529 402 560 565 544 ...
```

Explorative data analysis with graphics

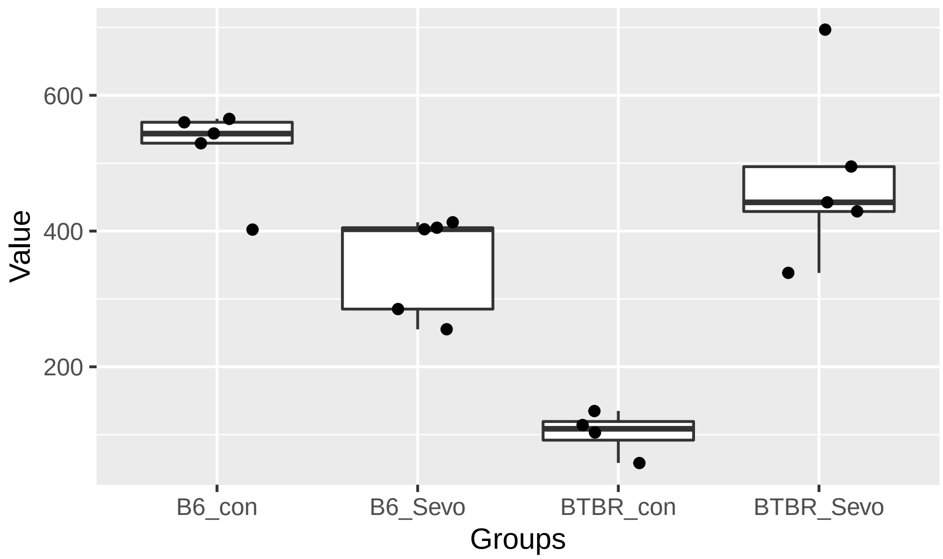

Easystat function developed by S. Park (available at https://rpubs.com/goodlebang)

Statistical Result

```
easystat(d1)
```

```
## 1. Normality assumption test by Shapiro_Wilk test is
##  p = 0.152
##  Normality assumption was not rejected
## 2. Equal variance test by Bartlett test is
##  p = 0.150
##  Equal variance assumption was not rejected
## 3. The result of anova is
##  p = 0.000
##  A statistically significant difference exist between groups
```

```
##   Tukey multiple comparisons of means
##     95% family-wise confidence level
##
## Fit: aov(formula = d1[, 3] ~ d1[, 2], data = d1)
##
## $`d1[, 2]`
##                          diff        lwr         upr     p adj
## B6_Sevo-B6_con     -168.02064 -328.04426   -7.997022 0.0380760
## BTBR_con-B6_con    -417.44916 -587.17984 -247.718484 0.0000199
## BTBR_Sevo-B6_con    -39.94338 -199.96700  120.080238 0.8878376
## BTBR_con-B6_Sevo   -249.42852 -419.15920  -79.697844 0.0035813
## BTBR_Sevo-B6_Sevo   128.07726  -31.94636  288.100878 0.1405862
## BTBR_Sevo-BTBR_con  377.50578  207.77510  547.236461 0.0000625
```

Fig 2 A. stage IV Data analysis using R

Boohwi Hong

Package install

Data import

Data structure

```
str(d1)
```

```
## 'data.frame':    19 obs. of  3 variables:
##  $ subject : int  1 2 3 4 5 6 7 8 9 10 ...
##  $ group   : chr  "B6_con" "B6_con" "B6_con" "B6_con" ...
##  $ stage.IV: num  1024 849 781 964 873 ...
```

Explorative data analysis with graphics

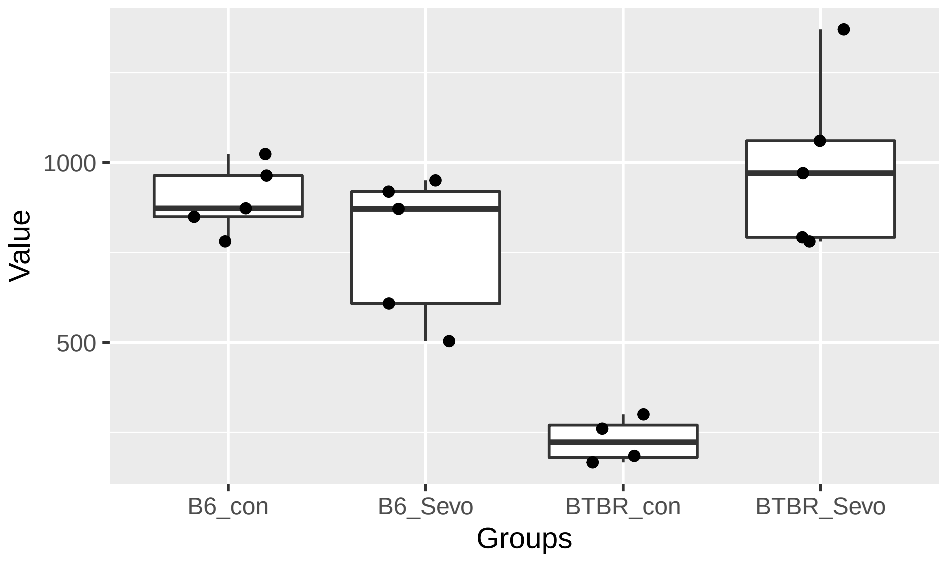

Easystat function developed by S. Park (available at https://rpubs.com/goodlebang)

Statistical Result

```
easystat(d1)
```

```
## 1. Normality assumption test by Shapiro_Wilk test is
##  p = 0.842
##  Normality assumption was not rejected
## 2. Equal variance test by Bartlett test is
##  p = 0.109
##  Equal variance assumption was not rejected
## 3. The result of anova is
##  p = 0.000
##  A statistically significant difference exist between groups
```

```
##   Tukey multiple comparisons of means
##     95% family-wise confidence level
##
## Fit: aov(formula = d1[, 3] ~ d1[, 2], data = d1)
##
## $`d1[, 2]`
##                          diff         lwr       upr     p adj
## B6_Sevo-B6_con     -127.4561  -440.93428  186.0221 0.6528094
## BTBR_con-B6_con    -669.9951 -1002.48890 -337.5012 0.0001818
## BTBR_Sevo-B6_con     96.8010  -216.67720  410.2792 0.8100846
## BTBR_con-B6_Sevo   -542.5390  -875.03282 -210.0451 0.0014412
## BTBR_Sevo-B6_Sevo   224.2571   -89.22112  537.7353 0.2100600
## BTBR_Sevo-BTBR_con  766.7961   434.30221 1099.2899 0.0000416
```

Fig 2 B. ATP5A Data analysis using R

Boohwi Hong

Package install

Data import

Data structure

```
str(d1)
```

```
## 'data.frame':    12 obs. of  3 variables:
##  $ subject: int  1 2 3 4 5 6 7 8 9 10 ...
##  $ group  : chr  "B6" "B6" "B6" "B6" ...
##  $ ATP5A  : num  1.03 1.016 0.995 0.959 0.129 ...
```

Explorative data analysis with graphics

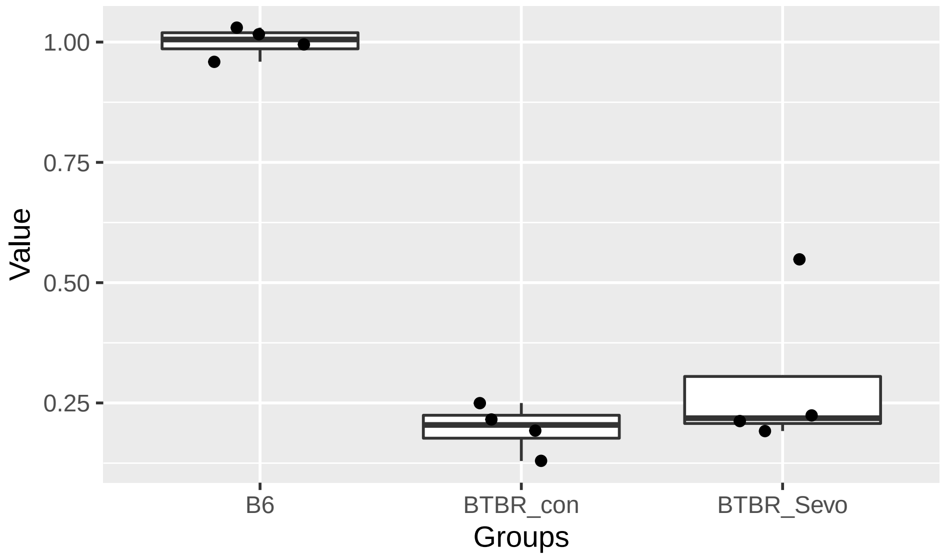

Easystat function developed by S. Park (available at https://rpubs.com/goodlebang)

Statistical Result

```
easystat(d1)
```

```
## 1. Normality assumption test by Shapiro_Wilk test is
##  p = 0.016
##  Normality assumption was rejected
## 2. The result of Kruskall_Wallis test:
##  p = 0.023
##  A statistically significant difference exist between groups

## Dunn (1964) Kruskal-Wallis multiple comparison

##   p-values adjusted with the Benjamini-Hochberg method.

##              Comparison          Z     P.unadj      P.adj
## 1        B6 - BTBR_con  2.5495098 0.01078745 0.03236235
## 2       B6 - BTBR_Sevo  2.1572775 0.03098405 0.04647608
## 3 BTBR_con - BTBR_Sevo -0.3922323 0.69488660 0.69488660
```

Fig 2 B. COX I Data analysis using R

Boohwi Hong

Package install

Data import

Data structure

```
str(d1)
```

```
## 'data.frame':    12 obs. of  3 variables:
##  $ subject: int  1 2 3 4 5 6 7 8 9 10 ...
##  $ group  : chr  "B6" "B6" "B6" "B6" ...
##  $ COX.I  : num  1.092 1.372 1.091 0.445 0.902 ...
```

Explorative data analysis with graphics

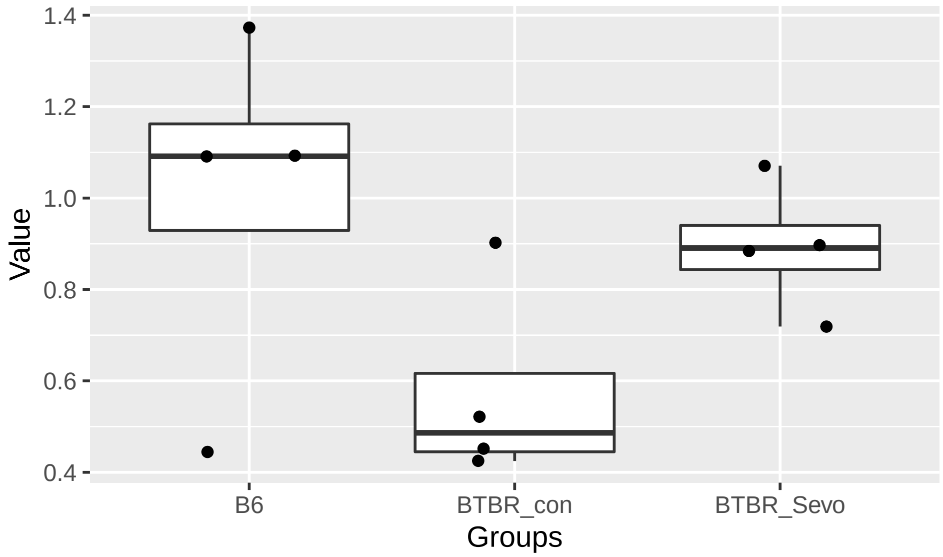

Easystat function developed by S. Park (available at https://rpubs.com/goodlebang)

Statistical Result

```
easystat(d1)
```

```
## 1. Normality assumption test by Shapiro_Wilk test is
##  p = 0.621
##  Normality assumption was not rejected
## 2. Equal variance test by Bartlett test is
##  p = 0.279
##  Equal variance assumption was not rejected
## 3. The result of anova is
##  p = 0.128
##  A statistically significant difference do not exist between groups
```

Fig 2 **B**. NDUFB8 Data analysis using R

Boohwi Hong

Package install

Data import

Data structure

```
str(d1)
```

```
## 'data.frame':    12 obs. of  3 variables:
##  $ subject: int  1 2 3 4 5 6 7 8 9 10 ...
##  $ group  : chr  "B6" "B6" "B6" "B6" ...
##  $ NDUFB8 : num  0.8696 0.8927 1.2247 1.0131 0.0138 ...
```

Explorative data analysis with graphics

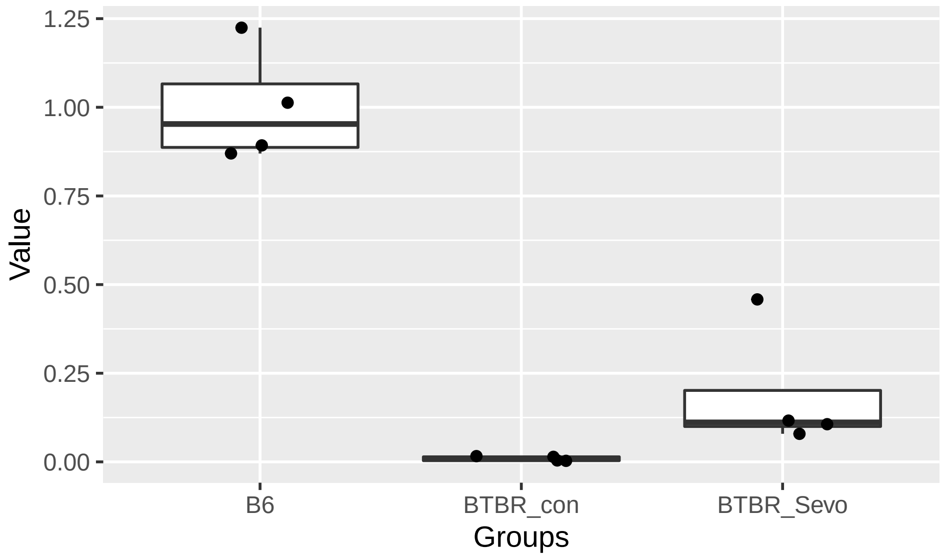

Easystat function developed by S. Park (available at https://rpubs.com/goodlebang)

Statistical Result

```
easystat(d1)
```

```
## 1. Normality assumption test by Shapiro_Wilk test is
##  p = 0.016
##  Normality assumption was rejected
## 2. The result of Kruskall_Wallis test:
##  p = 0.007
##  A statistically significant difference exist between groups
```

```
## Dunn (1964) Kruskal-Wallis multiple comparison
```

```
##   p-values adjusted with the Benjamini-Hochberg method.
```

```
##             Comparison         Z     P.unadj       P.adj
## 1        B6 - BTBR_con  3.137858 0.001701872 0.005105616
## 2       B6 - BTBR_Sevo  1.568929 0.116664465 0.116664465
## 3 BTBR_con - BTBR_Sevo -1.568929 0.116664465 0.174996697
```

Fig 2 B. SDHB Data analysis using R

Boohwi Hong

Package install

Data import

Data structure

```
str(d1)
```

```
## 'data.frame':    12 obs. of  3 variables:
##  $ subject: int  1 2 3 4 5 6 7 8 9 10 ...
##  $ group  : chr  "B6" "B6" "B6" "B6" ...
##  $ SDHB   : num  0.817 0.861 0.933 1.389 0.569 ...
```

Explorative data analysis with graphics

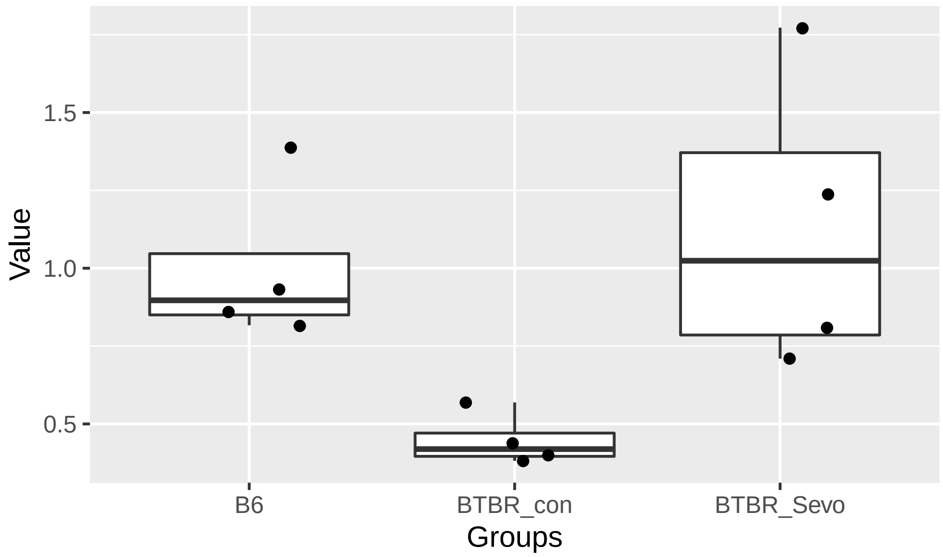

Easystat function developed by S. Park (available at https://rpubs.com/goodlebang)

Statistical Result

```
easystat(d1)
```

```
## 1. Normality assumption test by Shapiro_Wilk test is
##  p = 0.444
##  Normality assumption was not rejected
## 2. Equal variance test by Bartlett test is
##  p = 0.052
##  Equal variance assumption was not rejected
## 3. The result of anova is
##  p = 0.033
##  A statistically significant difference exist between groups
```

```
##   Tukey multiple comparisons of means
##     95% family-wise confidence level
##
## Fit: aov(formula = d1[, 3] ~ d1[, 2], data = d1)
##
## $`d1[, 2]`
##                         diff         lwr        upr     p adj
## BTBR_con-B6       -0.5527658 -1.18849706 0.08296556 0.0880298
## BTBR_Sevo-B6       0.1325871 -0.50314418 0.76831844 0.8327599
## BTBR_Sevo-BTBR_con  0.6853529  0.04962157 1.32108419 0.0355927
```

Fig 2 B. UQCRC2 Data analysis using R

Boohwi Hong

Package install

Data import

Data structure

```
str(d1)
```

```
## 'data.frame':    12 obs. of  3 variables:
##  $ subject: int  1 2 3 4 5 6 7 8 9 10 ...
##  $ group  : chr  "B6" "B6" "B6" "B6" ...
##  $ UQCRC2 : num  1.069 0.972 1.036 0.923 1.197 ...
```

Explorative data analysis with graphics

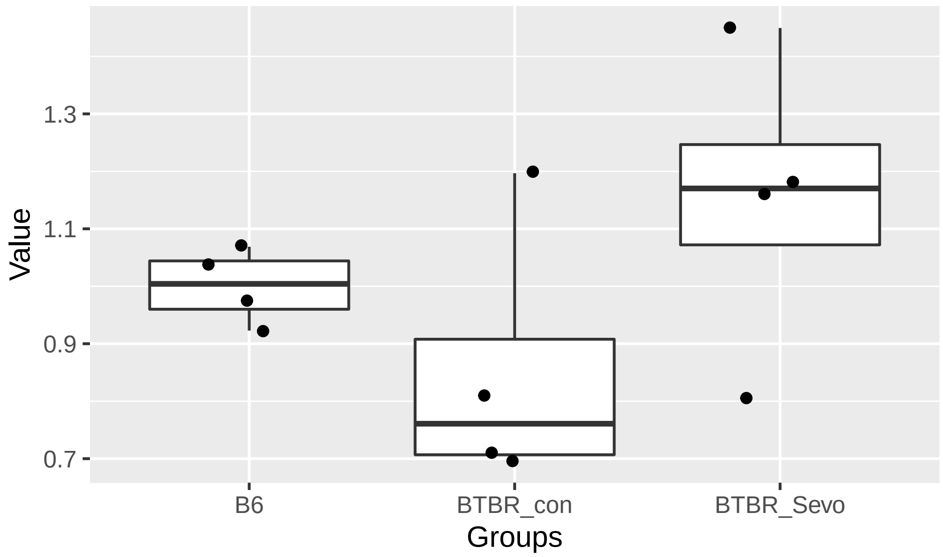

Easystat function developed by S. Park (available at https://rpubs.com/goodlebang)

Statistical Result

```
easystat(d1)
```

```
## 1. Normality assumption test by Shapiro_Wilk test is
##  p = 0.523
##  Normality assumption was not rejected
## 2. Equal variance test by Bartlett test is
##  p = 0.125
##  Equal variance assumption was not rejected
## 3. The result of anova is
##  p = 0.189
##  A statistically significant difference do not exist between groups
```

Fig 2 C. NDUFB8 Data analysis using R

Boohwi Hong

Package install

Data import

Data structure

```
str(d1)
```

```
## 'data.frame':    12 obs. of  3 variables:
##  $ subject: int  1 2 3 4 5 6 7 8 9 10 ...
##  $ group  : chr  "B6_con" "B6_con" "B6_con" "B6_con" ...
##  $ NDUFB8 : num  0.868 0.764 0.89 1.051 1.093 ...
```

Explorative data analysis with graphics

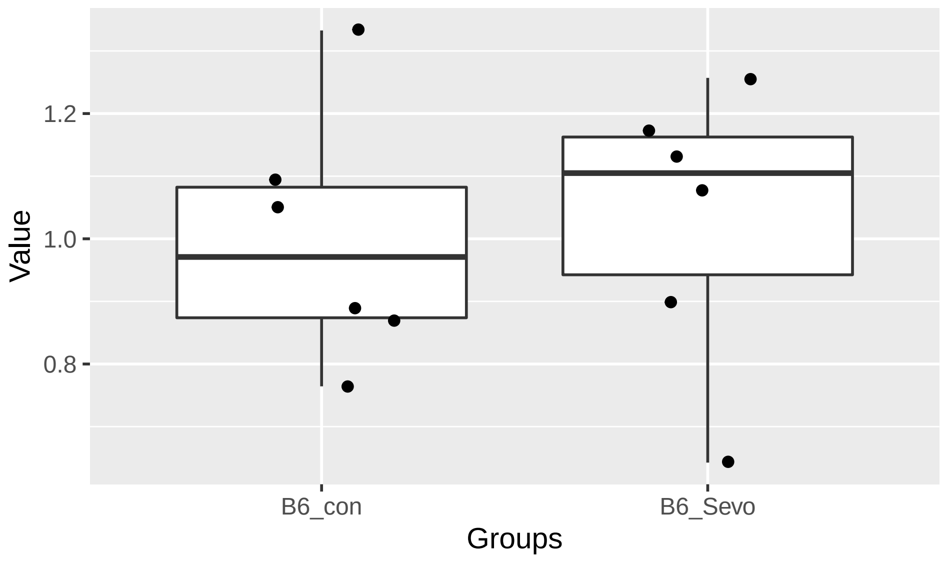

Easystat function developed by S. Park (available at https://rpubs.com/goodlebang)

Statistical Result

```
easystat(d1)
```

```
## 1. Normality assumption test by Shapiro_Wilk test is
##  p = 0.941
##  Normality assumption was not rejected
## 2. Equal variance test by Bartlett test is
##  p = 0.832
##  Equal variance assumption was not rejected
## 3. The result of anova is
##  p = 0.814
##  A statistically significant difference do not exist between groups
```

Fig 2 C. SDHB Data analysis using R

Boohwi Hong

Package install

Data import

Data structure

```
str(d1)
```

```
## 'data.frame':    12 obs. of  3 variables:
##  $ subject: int  1 2 3 4 5 6 7 8 9 10 ...
##  $ group  : chr  "B6_con" "B6_con" "B6_con" "B6_con" ...
##  $ SDHB   : num  0.828 0.874 1.041 1.054 1.139 ...
```

Explorative data analysis with graphics

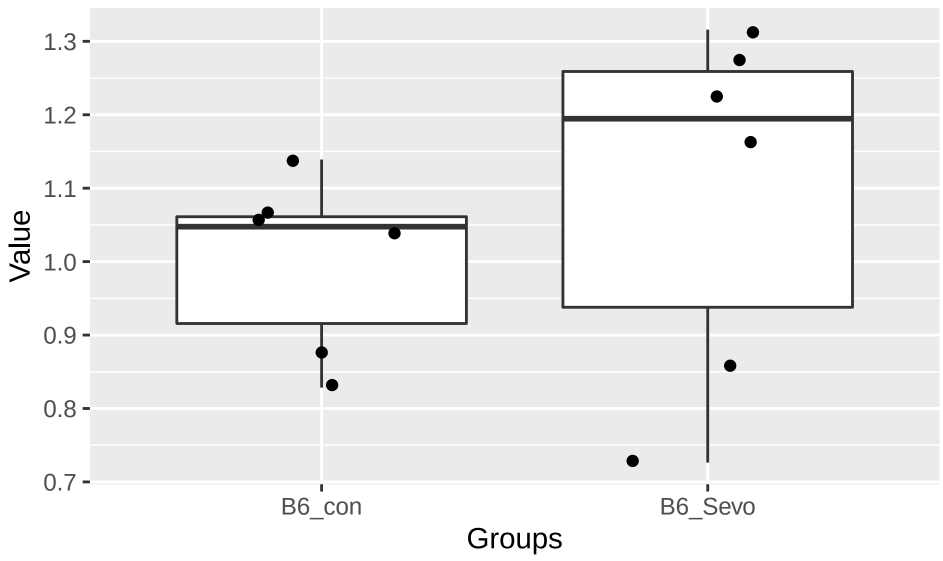

Easystat function developed by S. Park (available at https://rpubs.com/goodlebang)

Statistical Result

```
easystat(d1)
```

```
## 1. Normality assumption test by Shapiro_Wilk test is
##  p = 0.197
##  Normality assumption was not rejected
## 2. Equal variance test by Bartlett test is
##  p = 0.156
##  Equal variance assumption was not rejected
## 3. The result of anova is
##  p = 0.414
##  A statistically significant difference do not exist between groups
```

Fig 2 C. UQCRC2 Data analysis using R

Boohwi Hong

Package install

Data import

Data structure

```
str(d1)
```

```
## 'data.frame':    12 obs. of  3 variables:
##  $ subject: int  1 2 3 4 5 6 7 8 9 10 ...
##  $ group  : chr  "B6_con" "B6_con" "B6_con" "B6_con" ...
##  $ UQCRC2 : num  1.115 0.944 0.917 0.876 1.002 ...
```

Explorative data analysis with graphics

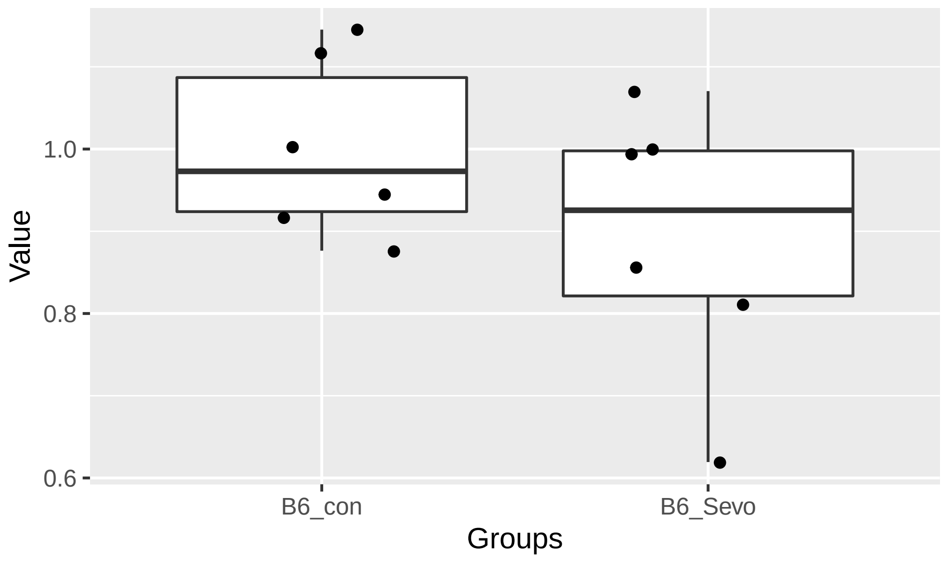

Easystat function developed by S. Park (available at https://rpubs.com/goodlebang)

Statistical Result

```
easystat(d1)
```

```
## 1. Normality assumption test by Shapiro_Wilk test is
##  p = 0.48
##  Normality assumption was not rejected
## 2. Equal variance test by Bartlett test is
##  p = 0.386
##  Equal variance assumption was not rejected
## 3. The result of anova is
##  p = 0.209
##  A statistically significant difference do not exist between groups
```

Fig 2 C. COX I Data analysis using R

Boohwi Hong

Package install

Data import

Data structure

```
str(d1)
```

```
## 'data.frame':    12 obs. of  3 variables:
##  $ subject: int  1 2 3 4 5 6 7 8 9 10 ...
##  $ group  : chr  "B6_con" "B6_con" "B6_con" "B6_con" ...
##  $ COX.I  : num  1.008 0.999 0.884 0.97 0.971 ...
```

Explorative data analysis with graphics

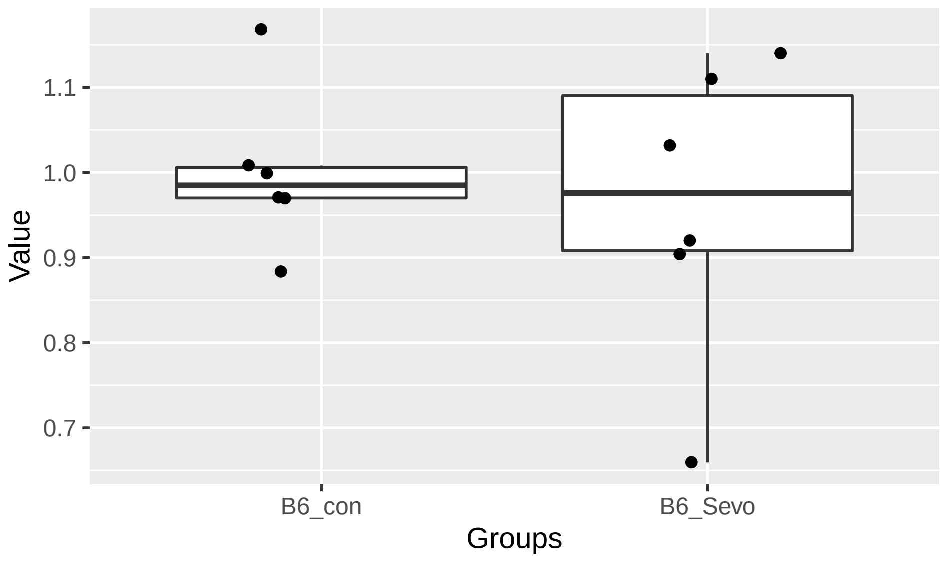

Easystat function developed by S. Park (available at https://rpubs.com/goodlebang)

Statistical Result

```
easystat(d1)
```

```
## 1. Normality assumption test by Shapiro_Wilk test is
##  p = 0.306
##  Normality assumption was not rejected
## 2. Equal variance test by Bartlett test is
##  p = 0.189
##  Equal variance assumption was not rejected
## 3. The result of anova is
##  p = 0.642
##  A statistically significant difference do not exist between groups
```

Fig 2 C. ATP5A Data analysis using R

Boohwi Hong

Package install

Data import

Data structure

```
str(d1)
```

```
## 'data.frame':    12 obs. of  3 variables:
##  $ subject: int  1 2 3 4 5 6 7 8 9 10 ...
##  $ group  : chr  "B6_con" "B6_con" "B6_con" "B6_con" ...
##  $ ATP5A  : num  0.504 0.945 1.124 1.198 1.127 ...
```

Explorative data analysis with graphics

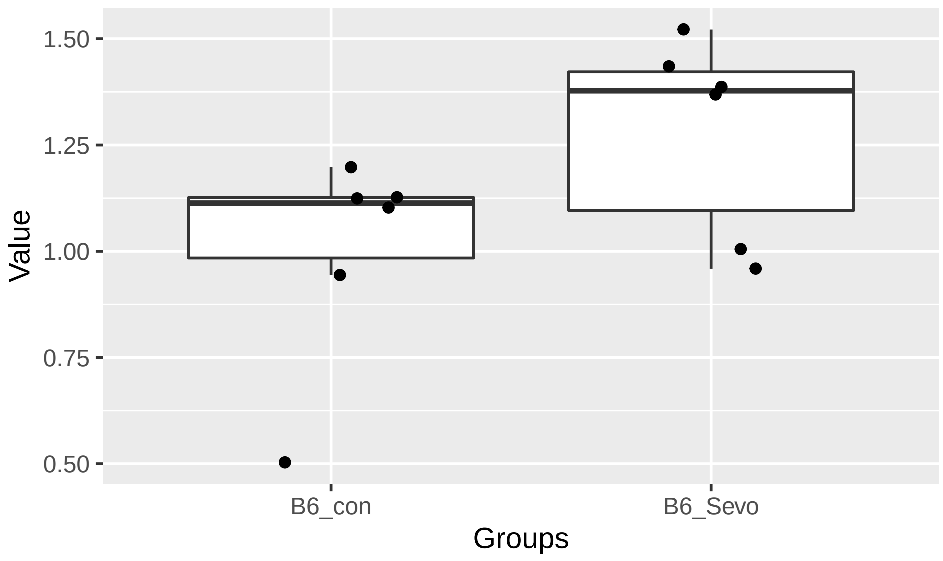

Easystat function developed by S. Park (available at https://rpubs.com/goodlebang)

Statistical Result

```
easystat(d1)
```

```
## 1. Normality assumption test by Shapiro_Wilk test is
##  p = 0.018
##  Normality assumption was rejected
## 2. The result of Kruskall_Wallis test:
##  p = 0.109
##  A statistically significant difference do not exist between groups
```

2.3 The results of statistical analysis

Figure 3

Fig 3 B. BDNF Data analysis using R

Boohwi Hong

Package install

Data import

Data structure

```
str(d1)
```

```
## 'data.frame':    12 obs. of  3 variables:
##  $ subject: int  1 2 3 4 5 6 7 8 9 10 ...
##  $ group  : chr  "B6" "B6" "B6" "B6" ...
##  $ BDNF   : num  0.69 0.962 1.262 1.086 0.536 ...
```

Explorative data analysis with graphics

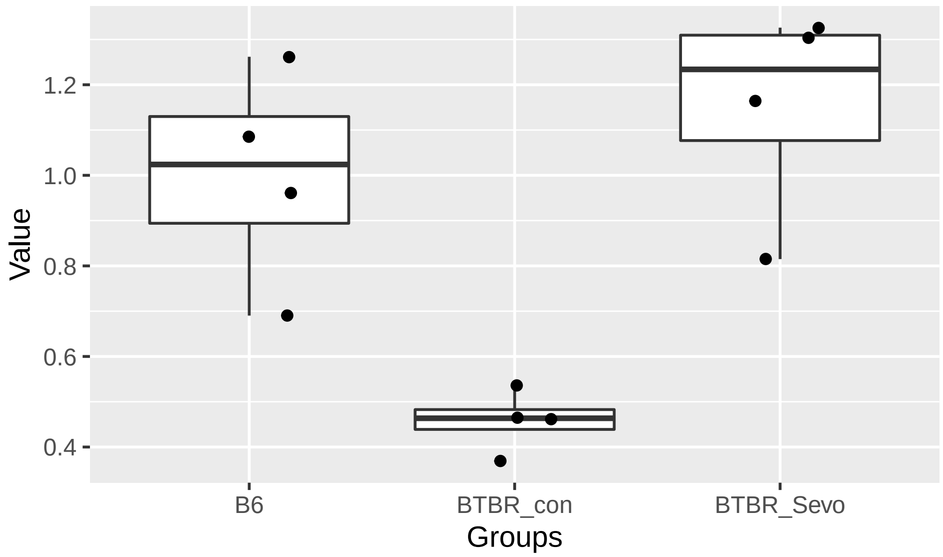

Easystat function developed by S. Park (available at https://rpubs.com/goodlebang)

Statistical Result

```
easystat(d1)
```

```
## 1. Normality assumption test by Shapiro_Wilk test is
##  p = 0.308
##  Normality assumption was not rejected
## 2. Equal variance test by Bartlett test is
##  p = 0.161
##  Equal variance assumption was not rejected
## 3. The result of anova is
##  p = 0.002
##  A statistically significant difference exist between groups
```

```
##   Tukey multiple comparisons of means
##     95% family-wise confidence level
##
## Fit: aov(formula = d1[, 3] ~ d1[, 2], data = d1)
##
## $`d1[, 2]`
##                         diff        lwr        upr     p adj
## BTBR_con-B6       -0.5419555 -0.9337947 -0.1501164 0.0096557
## BTBR_Sevo-B6       0.1522790 -0.2395602  0.5441181 0.5461483
## BTBR_Sevo-BTBR_con  0.6942345  0.3023954  1.0860737 0.0020543
```

Fig 3 B. p-TrkB_TrkB Data analysis using R

Boohwi Hong

Package install

Data import

Data structure

```
str(d1)
```

```
## 'data.frame':    12 obs. of  3 variables:
##  $ subject    : int  1 2 3 4 5 6 7 8 9 10 ...
##  $ group      : chr  "B6" "B6" "B6" "B6" ...
##  $ p.TrkB.TrkB: num  1.349 1.267 0.737 0.648 0.637 ...
```

Explorative data analysis with graphics

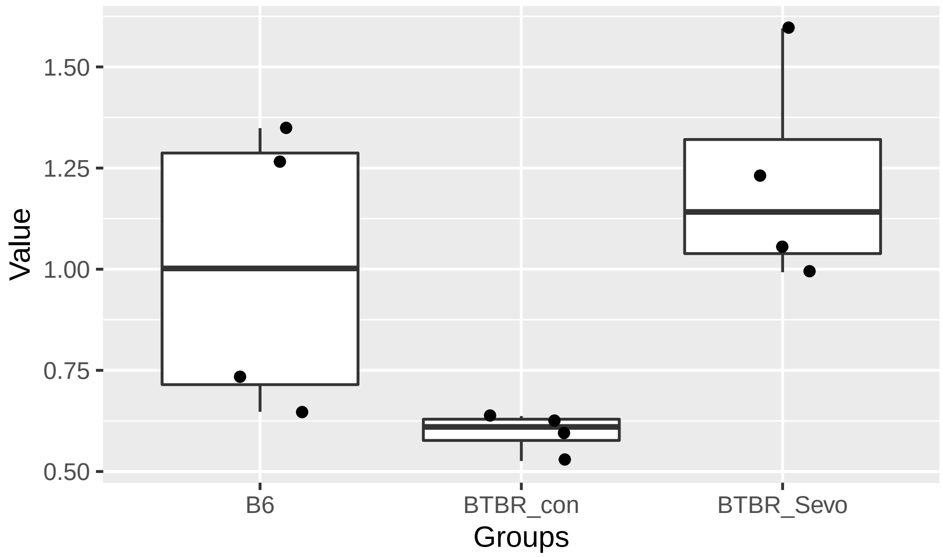

Easystat function developed by S. Park (available at https://rpubs.com/goodlebang)

Statistical Result

```
easystat(d1)
```

```
## 1. Normality assumption test by Shapiro_Wilk test is
##  p = 0.534
##  Normality assumption was not rejected
## 2. Equal variance test by Bartlett test is
##  p = 0.034
##  Equal variance assumption was rejected
## 3. The result of Welch ANOVA is
##  p = 0.022
##  A statistically significant difference exist between groups
```

```
##   Tukey multiple comparisons of means
##     95% family-wise confidence level
##
## Fit: aov(formula = d1[, 3] ~ d1[, 2])
##
## $`d1[, 2]`
##                          diff        lwr       upr     p adj
## BTBR_con-B6        -0.4041793 -0.9197210 0.1113625 0.1266878
## BTBR_Sevo-B6        0.2177229 -0.2978188 0.7332646 0.4936896
## BTBR_Sevo-BTBR_con  0.6219022  0.1063604 1.1374439 0.0204294
```

Fig 3.D BDNF Data analysis using R

Boohwi Hong

Package install

Data import

Data structure

```
str(d1)
```

```
## 'data.frame':    12 obs. of  3 variables:
##  $ subject: int  1 2 3 4 5 6 7 8 9 10 ...
##  $ group  : chr  "B6_con" "B6_con" "B6_con" "B6_con" ...
##  $ BDNF   : num  0.727 0.879 1.172 1.05 1.033 ...
```

Explorative data analysis with graphics

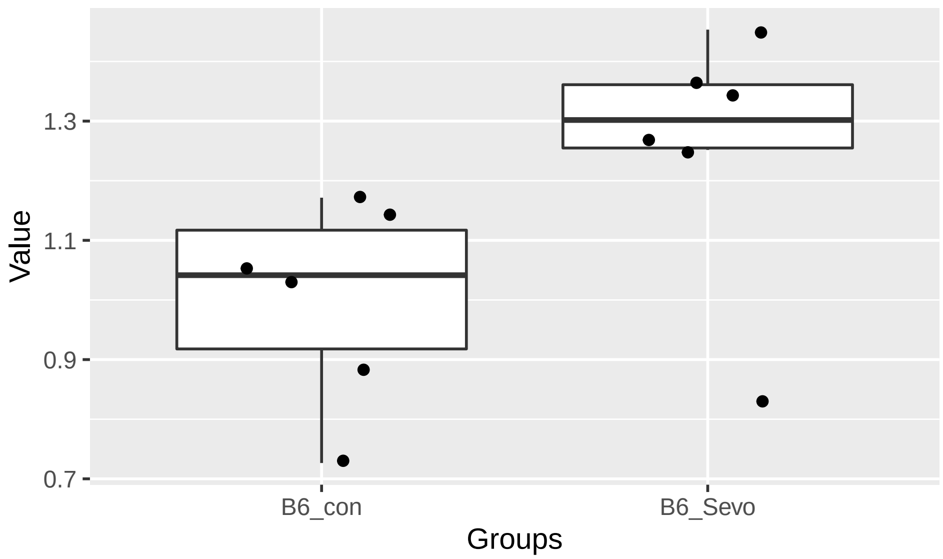

Easystat function developed by S. Park (available at https://rpubs.com/goodlebang)

Statistical Result

```
easystat(d1)
```

```
## 1. Normality assumption test by Shapiro_Wilk test is
##  p = 0.065
##  Normality assumption was not rejected
## 2. Equal variance test by Bartlett test is
##  p = 0.578
##  Equal variance assumption was not rejected
## 3. The result of anova is
##  p = 0.050
##  A statistically significant difference do not exist between groups
```

Fig 3.D pTrkBTrkB Data analysis using R

Boohwi Hong

Package install

Data import

Data structure

```
str(d1)
```

```
## 'data.frame':    12 obs. of  3 variables:
##  $ subject    : int  1 2 3 4 5 6 7 8 9 10 ...
##  $ group      : chr  "B6_con" "B6_con" "B6_con" "B6_con" ...
##  $ p.TrkB.TrkB: num  1.163 1.055 1.122 0.874 0.893 ...
```

Explorative data analysis with graphics

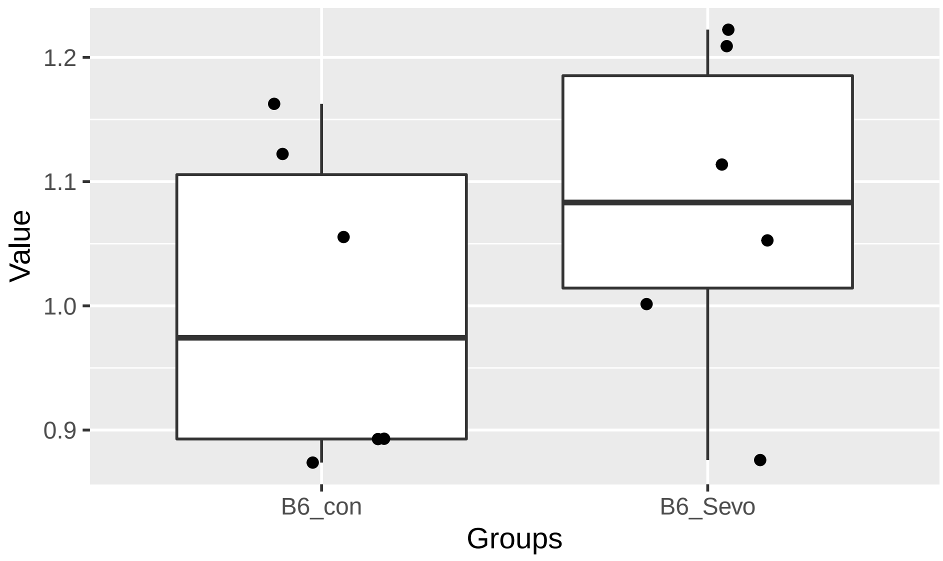

Easystat function developed by S. Park (available at https://rpubs.com/goodlebang)

Statistical Result

```r
easystat(d1)
```

```
## 1. Normality assumption test by Shapiro_Wilk test is
##  p = 0.312
##  Normality assumption was not rejected
## 2. Equal variance test by Bartlett test is
##  p = 0.967
##  Equal variance assumption was not rejected
## 3. The result of anova is
##  p = 0.317
##  A statistically significant difference do not exist between groups
```

2.4 The results of statistical analysis

Figure 4

Fig 4 A. B6_con Data analysis using R

Boohwi Hong

Package install

Data import

Data structure

```
str(d1)
```

```
## 'data.frame':    20 obs. of  3 variables:
##  $ subject: int  1 2 3 4 5 6 7 8 9 10 ...
##  $ group  : chr  "ob" "ob" "ob" "ob" ...
##  $ time   : num  212 217 189 175 172 ...
```

Explorative data analysis with graphics

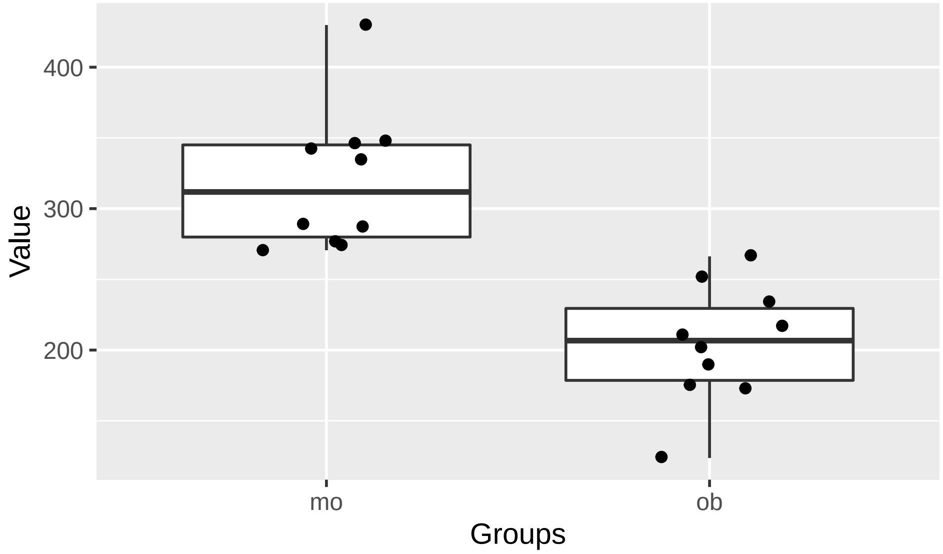

Easystat function developed by S. Park (available at https://rpubs.com/goodlebang)

Statistical Result

```
easystat(d1)
```

```
## 1. Normality assumption test by Shapiro_Wilk test is
##  p = 0.687
##  Normality assumption was not rejected
## 2. Equal variance test by Bartlett test is
##  p = 0.607
##  Equal variance assumption was not rejected
## 3. The result of anova is
##  p = 0.000
##  A statistically significant difference exist between groups
```

```
##   Tukey multiple comparisons of means
##     95% family-wise confidence level
##
## Fit: aov(formula = d1[, 3] ~ d1[, 2], data = d1)
##
## $‘d1[, 2]‘
##           diff       lwr       upr    p adj
## ob-mo -115.6225 -159.0115 -72.23355 2.59e-05
```

Fig 4 A. B6_Sevo Data analysis using R

Boohwi Hong

Package install

Data import

Data structure

```
str(d1)
```

```
## 'data.frame':    18 obs. of  3 variables:
##  $ subject: int  1 2 3 4 5 6 7 8 9 10 ...
##  $ group  : chr  "ob" "ob" "ob" "ob" ...
##  $ time   : num  179 217 161 129 249 ...
```

Explorative data analysis with graphics

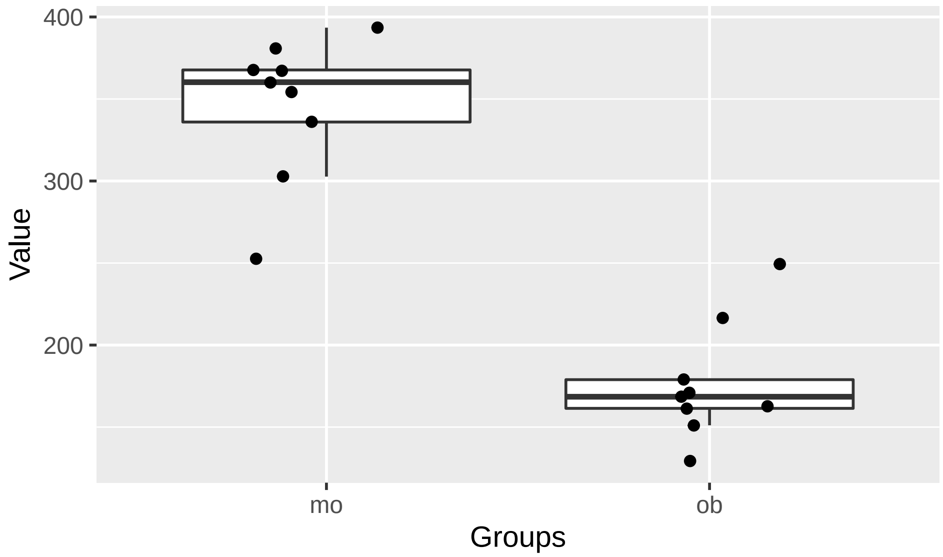

Easystat function developed by S. Park (available at https://rpubs.com/goodlebang)

Statistical Result

```
easystat(d1)
```

```
## 1. Normality assumption test by Shapiro_Wilk test is
##  p = 0.935
##  Normality assumption was not rejected
## 2. Equal variance test by Bartlett test is
##  p = 0.583
##  Equal variance assumption was not rejected
## 3. The result of anova is
##  p = 0.000
##  A statistically significant difference exist between groups
```

```
##   Tukey multiple comparisons of means
##     95% family-wise confidence level
##
## Fit: aov(formula = d1[, 3] ~ d1[, 2], data = d1)
##
## $`d1[, 2]`
##            diff       lwr       upr p adj
## ob-mo -169.5502 -209.5739 -129.5266 1e-07
```

Fig 4 A. BTBR_con Data analysis using R

Boohwi Hong

Package install

Data import

Data structure

```
str(d1)
```

```
## 'data.frame':    20 obs. of  3 variables:
##  $ subject: int  1 2 3 4 5 6 7 8 9 10 ...
##  $ group  : chr  "ob" "ob" "ob" "ob" ...
##  $ time   : num  213 348 404 285 210 ...
```

Explorative data analysis with graphics

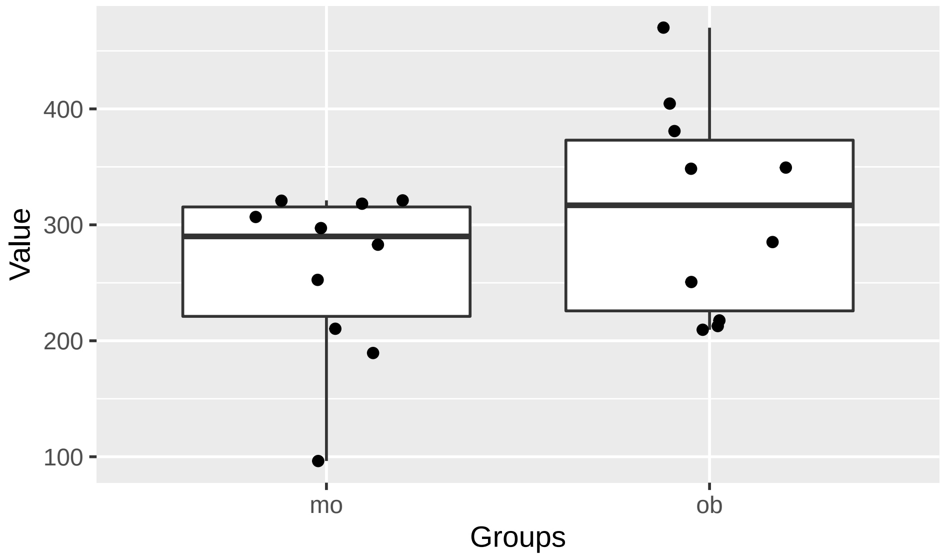

Easystat function developed by S. Park (available at https://rpubs.com/goodlebang)

Statistical Result

```
easystat(d1)
```

```
## 1. Normality assumption test by Shapiro_Wilk test is
##  p = 0.529
##  Normality assumption was not rejected
## 2. Equal variance test by Bartlett test is
##  p = 0.543
##  Equal variance assumption was not rejected
## 3. The result of anova is
##  p = 0.168
##  A statistically significant difference do not exist between groups
```

Fig 4 A. BTBR_Sevo Data analysis using R

Boohwi Hong

Package install

Data import

Data structure

```
str(d1)
```

```
## 'data.frame':    20 obs. of  3 variables:
##  $ subject: int  1 2 3 4 5 6 7 8 9 10 ...
##  $ group  : chr  "ob" "ob" "ob" "ob" ...
##  $ time   : num  169 223 185 282 210 ...
```

Explorative data analysis with graphics

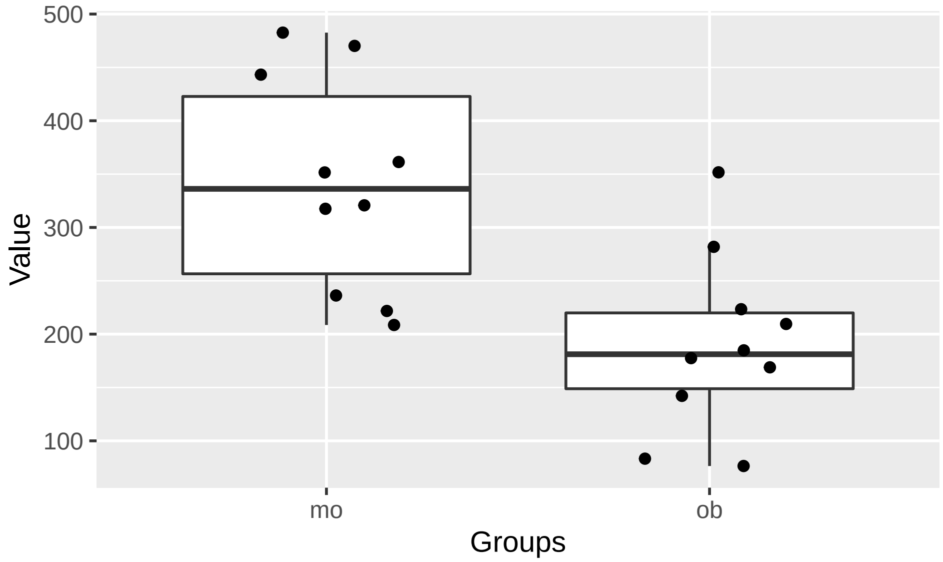

Easystat function developed by S. Park (available at https://rpubs.com/goodlebang)

Statistical Result

```
easystat(d1)
```

```
## 1. Normality assumption test by Shapiro_Wilk test is
##  p = 0.253
##  Normality assumption was not rejected
## 2. Equal variance test by Bartlett test is
##  p = 0.592
##  Equal variance assumption was not rejected
## 3. The result of anova is
##  p = 0.002
##  A statistically significant difference exist between groups
```

```
##   Tukey multiple comparisons of means
##     95% family-wise confidence level
##
## Fit: aov(formula = d1[, 3] ~ d1[, 2], data = d1)
##
## $`d1[, 2]`
##            diff       lwr       upr     p adj
## ob-mo -151.4206 -238.5126 -64.32863 0.0018206
```

Fig 4 B. 3 chamber preferenc index Data analysis using R

Boohwi Hong

Package install

Data import

Data structure

```
str(d1)
```

```
## 'data.frame':    39 obs. of  6 variables:
##  $ subject   : int  1 2 3 4 5 6 7 8 9 10 ...
##  $ group     : chr  "B6_con" "B6_con" "B6_con" "B6_con" ...
##  $ Preference: num  0.134 0.14 0.287 0.331 0.335 ...
##  $ X         : logi  NA NA NA NA NA NA ...
##  $ X.1       : logi  NA NA NA NA NA NA ...
##  $ X.2       : logi  NA NA NA NA NA NA ...
```

Explorative data analysis with graphics

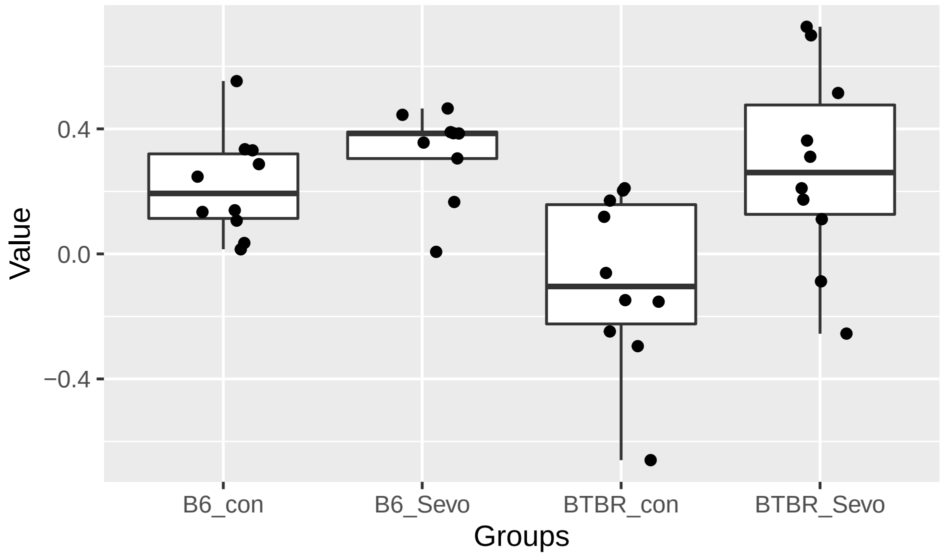

Easystat function developed by S. Park (available at https://rpubs.com/goodlebang)

Statistical Result

```
easystat(d1)
```

```
## 1. Normality assumption test by Shapiro_Wilk test is
##  p = 0.609
##  Normality assumption was not rejected
## 2. Equal variance test by Bartlett test is
##  p = 0.086
##  Equal variance assumption was not rejected
## 3. The result of anova is
##  p = 0.002
##  A statistically significant difference exist between groups
```

```
##   Tukey multiple comparisons of means
##     95% family-wise confidence level
##
## Fit: aov(formula = d1[, 3] ~ d1[, 2], data = d1)
##
## $`d1[, 2]`
##                          diff         lwr         upr     p adj
## B6_Sevo-B6_con      0.1044712 -0.19249063  0.40143303 0.7788822
## BTBR_con-B6_con    -0.3045935 -0.59363492 -0.01555208 0.0356772
## BTBR_Sevo-B6_con    0.0580961 -0.23094532  0.34713752 0.9480607
## BTBR_con-B6_Sevo   -0.4090647 -0.70602653 -0.11210287 0.0037667
## BTBR_Sevo-B6_Sevo  -0.0463751 -0.34333693  0.25058673 0.9744885
## BTBR_Sevo-BTBR_con  0.3626896  0.07364818  0.65173102 0.0091743
```

Fig 4 C. Grooming Data analysis using R

Boohwi Hong

Package install

Data import

Data structure

```
str(d1)
```

```
## 'data.frame':    39 obs. of  3 variables:
##  $ subject : int  1 2 3 4 5 6 7 8 9 10 ...
##  $ group   : chr  "B6_con" "B6_con" "B6_con" "B6_con" ...
##  $ grooming: int  44 52 108 55 12 8 97 46 20 35 ...
```

Explorative data analysis with graphics

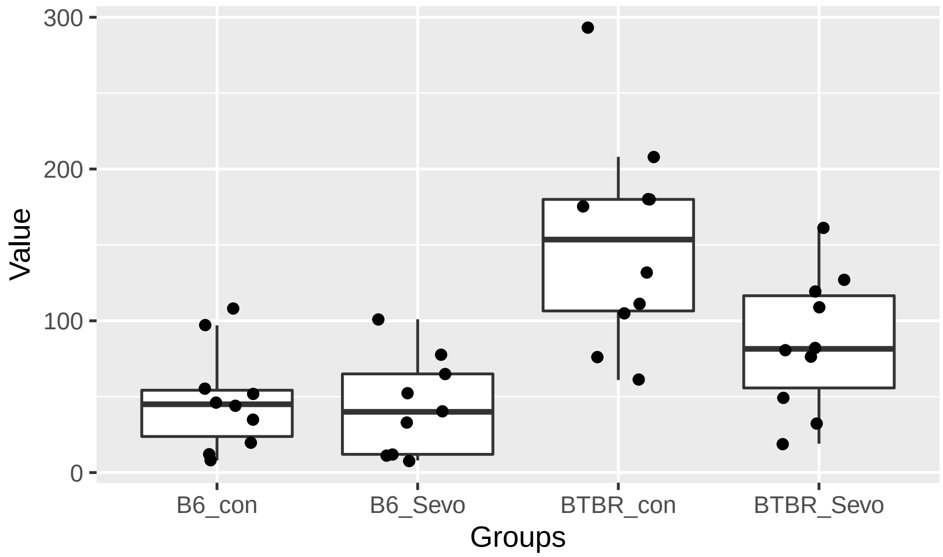

Easystat function developed by S. Park (available at https://rpubs.com/goodlebang)

Statistical Result

```
easystat(d1)
```

```
## 1. Normality assumption test by Shapiro_Wilk test is
##  p = 0.485
##  Normality assumption was not rejected
## 2. Equal variance test by Bartlett test is
##  p = 0.078
##  Equal variance assumption was not rejected
## 3. The result of anova is
##  p = 0.000
##  A statistically significant difference exist between groups
```

```
##   Tukey multiple comparisons of means
##     95% family-wise confidence level
##
## Fit: aov(formula = d1[, 3] ~ d1[, 2], data = d1)
##
## $`d1[, 2]`
##                          diff        lwr        upr     p adj
## B6_Sevo-B6_con      -3.255556  -62.38136  55.870250 0.9988096
## BTBR_con-B6_con    104.400000   46.85117 161.948834 0.0001259
## BTBR_Sevo-B6_con    37.800000  -19.74883  95.348834 0.3038404
## BTBR_con-B6_Sevo   107.655556   48.52975 166.781361 0.0001194
## BTBR_Sevo-B6_Sevo   41.055556  -18.07025 100.181361 0.2580747
## BTBR_Sevo-BTBR_con -66.600000 -124.14883  -9.051166 0.0180463
```

2.5 The results of statistical analysis
（ Supplementary Figure 1）

Data analysis using R

Boohwi Hong

```
## Present data is ** S Fig 1. B6.csv **
##
## **  Data structure  **
## 'data.frame':    26 obs. of  3 variables:
##  $ subject: int  1 2 3 4 5 6 7 8 9 10 ...
##  $ group  : chr  "ob" "ob" "ob" "ob" ...
##  $ time   : num  74.5 30.6 50.1 36.5 61 ...
##
## ** Explorative data analysis with graphics**
```

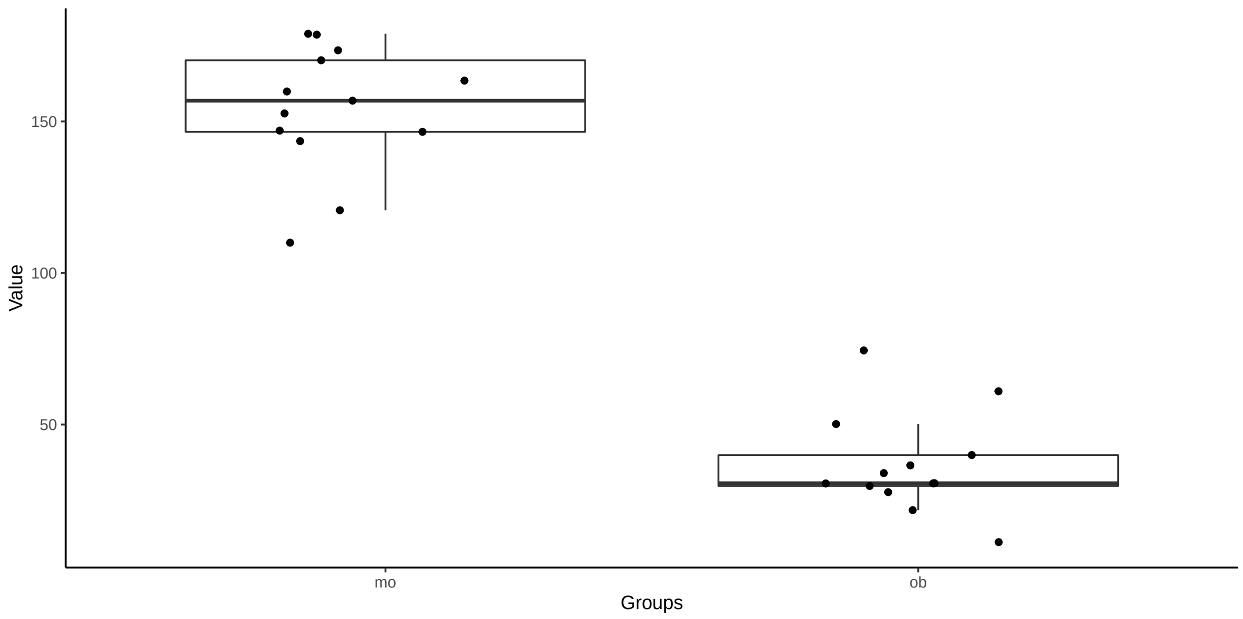


```
## 1. Normality assumption test by Shapiro_Wilk test is
##  p = 0.593
##  Normality assumption was not rejected
## 2. Equal variance test by Bartlett test is
##  p = 0.439
##  Equal variance assumption was not rejected
## 3. The result of anova is
##  p = 0.000
##  A statistically significant difference exist between groups
##
##   Tukey multiple comparisons of means
##     95% family-wise confidence level
##
```

```
## Fit: aov(formula = d1[, 3] ~ d1[, 2], data = d1)
##
## $`d1[, 2]`
##            diff       lwr       upr p adj
## ob-mo -117.1846 -132.4979 -101.8713     0
```

Data analysis using R

Boohwi Hong

```
## Present data is ** S Fig 1. BTBR.csv **
##
## **  Data structure  **
## 'data.frame':    34 obs. of  3 variables:
##  $ subject: int  1 2 3 4 5 6 7 8 9 10 ...
##  $ group  : chr  "ob" "ob" "ob" "ob" ...
##  $ time   : num  30.5 98.7 11.6 63.1 28.4 ...
##
## ** Explorative data analysis with graphics**
```

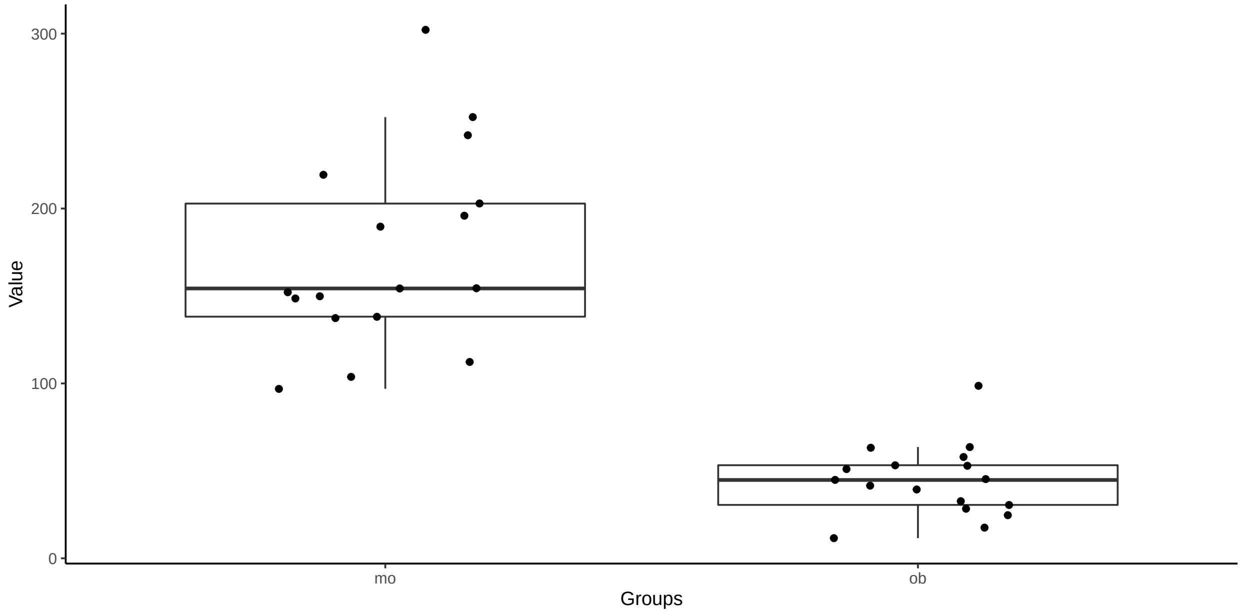


```
## 1. Normality assumption test by Shapiro_Wilk test is
##  p = 0.089
##  Normality assumption was not rejected
## 2. Equal variance test by Bartlett test is
##  p = 0.000
##  Equal variance assumption was rejected
## 3. The result of Welch ANOVA is
##  p = 0.000
##  A statistically significant difference exist between groups
##
##   Tukey multiple comparisons of means
##     95% family-wise confidence level
##
```

```
## Fit: aov(formula = d1[, 3] ~ d1[, 2])
##
## $`d1[, 2]`
##            diff       lwr       upr p adj
## ob-mo -129.1218 -158.7186 -99.52494     0
```

Data analysis using R

Boohwi Hong

```
## Present data is ** S Fig 1. BTBR_Sevoflurane.csv **
##
## **  Data structure  **
## 'data.frame':    32 obs. of  3 variables:
##  $ subject: int  1 2 3 4 5 6 7 8 9 10 ...
##  $ group  : chr  "ob" "ob" "ob" "ob" ...
##  $ time   : num  20 42.5 29.4 99 54.7 ...
##
## ** Explorative data analysis with graphics**
```

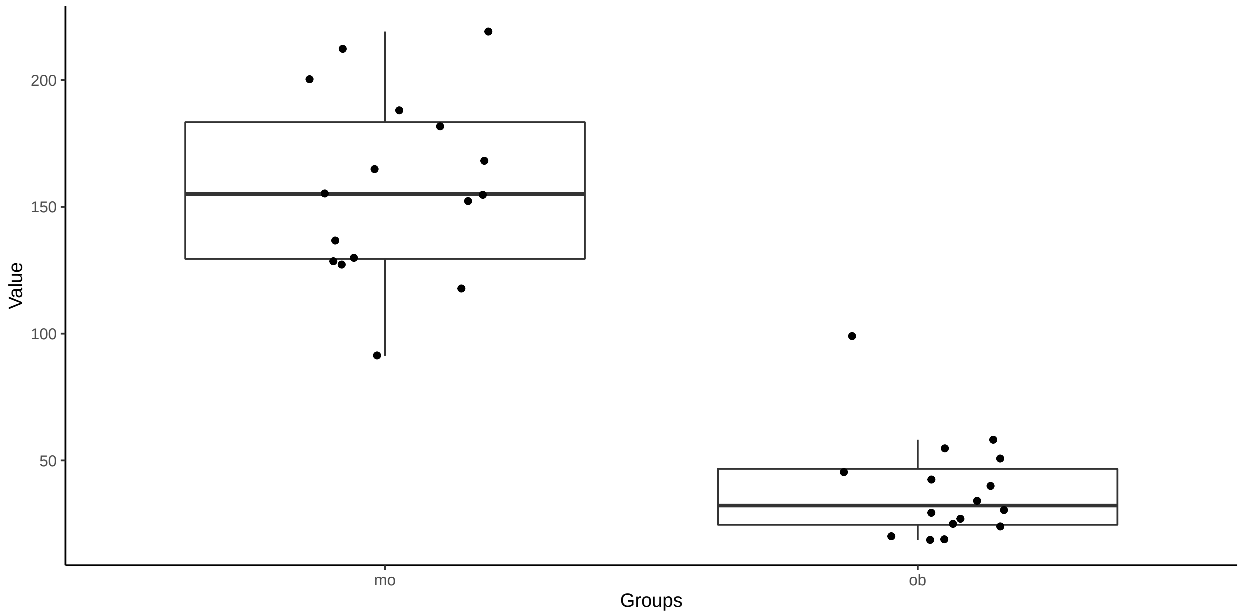


```
## 1. Normality assumption test by Shapiro_Wilk test is
##  p = 0.356
##  Normality assumption was not rejected
## 2. Equal variance test by Bartlett test is
##  p = 0.038
##  Equal variance assumption was rejected
## 3. The result of Welch ANOVA is
##  p = 0.000
##  A statistically significant difference exist between groups
##
##   Tukey multiple comparisons of means
##     95% family-wise confidence level
##
```

```
## Fit: aov(formula = d1[, 3] ~ d1[, 2])
##
## $`d1[, 2]`
##            diff       lwr       upr p adj
## ob-mo -119.4069 -140.5033 -98.31042     0
```
